# Loss of ALS2/Alsin Exacerbates Motor Dysfunction in a SOD1^H46R^-Expressing Mouse ALS Model by Disturbing Endolysosomal Trafficking

**DOI:** 10.1371/journal.pone.0009805

**Published:** 2010-03-22

**Authors:** Shinji Hadano, Asako Otomo, Ryota Kunita, Kyoko Suzuki-Utsunomiya, Akira Akatsuka, Masato Koike, Masashi Aoki, Yasuo Uchiyama, Yasuto Itoyama, Joh-E Ikeda

**Affiliations:** 1 Neurodegenerative Diseases Research Centre, Graduate School of Medicine, Tokai University, Isehara, Kanagawa, Japan; 2 Department of Molecular Life Sciences, Tokai University School of Medicine, Isehara, Kanagawa, Japan; 3 The Institute of Medical Sciences, Tokai University, Isehara, Kanagawa, Japan; 4 Teaching and Research Support Center, Tokai University, Isehara, Kanagawa, Japan; 5 Department of Cell Biology and Neuroscience, Juntendo University Graduate School of Medicine, Bunkyo-ku, Tokyo, Japan; 6 Department of Neurology, Tohoku University Graduate School of Medicine, Sendai, Miyagi, Japan; 7 Apoptosis Research Centre, Children Hospital of Eastern Ontario, Ottawa, Ontario, Canada; Brigham and Women's Hospital, Harvard Medical School, United States of America

## Abstract

**Background:**

ALS2/alsin is a guanine nucleotide exchange factor for the small GTPase Rab5 and involved in macropinocytosis-associated endosome fusion and trafficking, and neurite outgrowth. ALS2 deficiency accounts for a number of juvenile recessive motor neuron diseases (MNDs). Recently, it has been shown that ALS2 plays a role in neuroprotection against MND-associated pathological insults, such as toxicity induced by mutant Cu/Zn superoxide dismutase (SOD1). However, molecular mechanisms underlying the relationship between ALS2-associated cellular function and its neuroprotective role remain unclear.

**Methodology/Principal Findings:**

To address this issue, we investigated the molecular and pathological basis for the phenotypic modification of mutant SOD1-expressing mice by ALS2 loss. Genetic ablation of *Als2* in *SOD1^H46R^*, but not *SOD1^G93A^*, transgenic mice aggravated the mutant SOD1-associated disease symptoms such as body weight loss and motor dysfunction, leading to the earlier death. Light and electron microscopic examinations revealed the presence of degenerating and/or swollen spinal axons accumulating granular aggregates and autophagosome-like vesicles in early- and even pre-symptomatic *SOD1^H46R^* mice. Further, enhanced accumulation of insoluble high molecular weight SOD1, poly-ubiquitinated proteins, and macroautophagy-associated proteins such as polyubiquitin-binding protein p62/SQSTM1 and a lipidated form of light chain 3 (LC3-II), emerged in ALS2-deficient *SOD1^H46R^* mice. Intriguingly, ALS2 was colocalized with LC3 and p62, and partly with SOD1 on autophagosome/endosome hybrid compartments, and loss of ALS2 significantly lowered the lysosome-dependent clearance of LC3 and p62 in cultured cells.

**Conclusions/Significance:**

Based on these observations, although molecular basis for the distinctive susceptibilities to ALS2 loss in different mutant SOD1-expressing ALS models is still elusive, disturbance of the endolysosomal system by ALS2 loss may exacerbate the SOD1^H46R^-mediated neurotoxicity by accelerating the accumulation of immature vesicles and misfolded proteins in the spinal cord. We propose that ALS2 is implicated in endolysosomal trafficking through the fusion between endosomes and autophagosomes, thereby regulating endolysosomal protein degradation *in vivo*.

## Introduction

Amyotrophic lateral sclerosis (ALS) is a heterogeneous group of progressive neurodegenerative disorders characterized by a selective loss of upper motor neurons (UMN) in the cerebral cortex and lower motor neurons (LMN) in the brainstem and spinal cord [1]. While most of the cases reported are sporadic, 5–10% are familial. Thus far, more than 10 ALS-associated loci have been assigned, and several causative genes, including *SOD1* (ALS1), *ALS2*, *SETX* (ALS4), *SPATACSIN*/*SPG11* (ALS5), *FUS* (ALS6), *VAPB* (ALS8), *ANG* (ALS9), and *TARDBP* (ALS10) have been identified and characterized [1], [2], [3].

*ALS2* is a causative gene for a juvenile autosomal recessive form of motor neuron diseases (MNDs) [4], [5], [6], including amyotrophic lateral sclerosis 2 (ALS2) [7] (OMIM 205100), juvenile primary lateral sclerosis (PLSJ) [8] (OMIM 606353), and infantile-onset ascending hereditary spastic paralysis (IAHSP) [9] (OMIM 607225). These disorders are characterized by ascending degeneration of UMN with or without LMN involvement. A total of 19 independent *ALS2* mutations from 17 families have been reported [4], [5], [6], [10], [11], [12], [13], [14]. They are predicted to result in either premature termination of translation or substitution of an evolutionarily conserved amino acid for the *ALS2*-coded protein, ALS2 or alsin, leading to loss of its function. ALS2 is a guanine nucleotide exchange factor for the small GTPase Rab5 [15] and involves in macropinocytosis-associated endosome trafficking and fusion [16], [17], and neurite outgrowth [17], [18], [19]. Loss of these functions accounts for motor dysfunction and axonal degeneration in the *ALS2*-linked MNDs. Logically designed mouse studies could provide persuasive evidence that loss of ALS2 triggers motor neuron degeneration. However, mice lacking ALS2 cannot recapitulate the complex disease phenotypes, despite the subclinical levels of motor dysfunction and axonal degeneration in aged animals [6], [20], [21]. Thus, although there are potentially important clinical implications of these observations, the physiological functions of ALS2 and the molecular mechanisms underlying the motor dysfunction resulting from ALS2 deficiency remain to be clarified.

Most efforts to delineate ALS/MND pathogenesis have converged on the mutations in *SOD1*, encoding Cu/Zn superoxide dismutase (SOD1), which accounts for most prevalent form of the autosomal dominant familial ALS [22]. More than 120 different *SOD1* mutations have been identified (http://alsod.iop.kcl.ac.uk/als), and several transgenic mouse lines expressing disease causative SOD1 mutants have been generated and thoroughly characterized [23]. Nonetheless, no consensus has yet emerged as to how SOD1 mutations lead to selective death of motor neurons, except that multiple toxicity pathways including oxidative stress, endoplasmic reticulum (ER) stress, excitotoxicity, mitochondrial dysfunction, neural inflammation, protein misfolding and accumulation, and dysfunctional intracellular trafficking, are implicated in the pathogenesis of ALS/MNDs [1], [24].

Recently, it has been reported that overexpression of ALS2 protects cultured motor neuronal cells from toxicity induced by mutant SOD1 [25]. Further, loss of ALS2 renders neurons more susceptible to excitotoxicity [26], while cell death induced by neurotoxic stimuli, such as *N*-methyl-D-aspartate, is significantly suppressed by overexpression of ALS2 [27]. Moreover, *Als2*-null mice are slightly vulnerable to oxidative stress [28]. These observations imply a neuroprotective function of ALS2 against ALS/MND-associated pathological insults. On the other hand, *in vivo* studies have demonstrated that loss of ALS2 does not affect the motor neuron degeneration and survival of *SOD1^G93A^* mice [29], [30], which does not support the functional interaction between ALS2 and mutant SOD1-mediated toxicity *in vivo*. However, with the use of only a single mutant SOD1 transgenic line, such notions still remain inconclusive. Rather, it is possible that the extremely rapid progression of motor dysfunction observed in high-copy number *SOD1^G93A^* mice could overwhelm the modest symptoms by the ALS2 deficiency [29].

To clarify these issues, we used *SOD1^H46R^* mice, which exhibit a widespread axonal degeneration with slowly progressive motor neuron degeneration in the spinal cord [31] instead of *SOD1^G93A^* mice, and generated *SOD1^H46R^* mice on an *Als2*-null background. *SOD1^H46R^* mutation accounts for a mild form of familial ALS that was originally identified in Japanese kindred [32]. We here revealed that loss of ALS2 exacerbated the SOD1^H46R^-associated disease symptoms in mice, and identified ALS2 as a novel regulator for the endolysosomal system. Our findings suggest that ALS2 plays a role in the maturation of autophagosomes through the endosome-autophagosome fusion, thereby regulating endolysosomal trafficking *in vivo*.

## Results

### Loss of ALS2 results in a shorter lifespan in *SOD1^H46R^* mice

To investigate the effect of ALS2 expression on the pathogenesis for mutant SOD1-mediated MNDs, we generated congenic lines (C57BL/6N) with 6 different genotypes; *Als2* ^+/+^ (wild-type), *Als2* ^+/−^, *Als2* ^−/−^, *Als2* ^+/+^;*SOD1^H46R^*, *Als2* ^+/−^;*SOD1^H46R^*, and *Als2* ^−/−^;*SOD1^H46R^*. The mutant alleles were transmitted in the expected Mendelian ratio (Table S1). A copy number of the *SOD1^H46R^* transgene (∼20 copies) [31], which affected the disease severity [33], remained unchanged in the course of these mating schemes (Figure S1).

**Figure 1:**
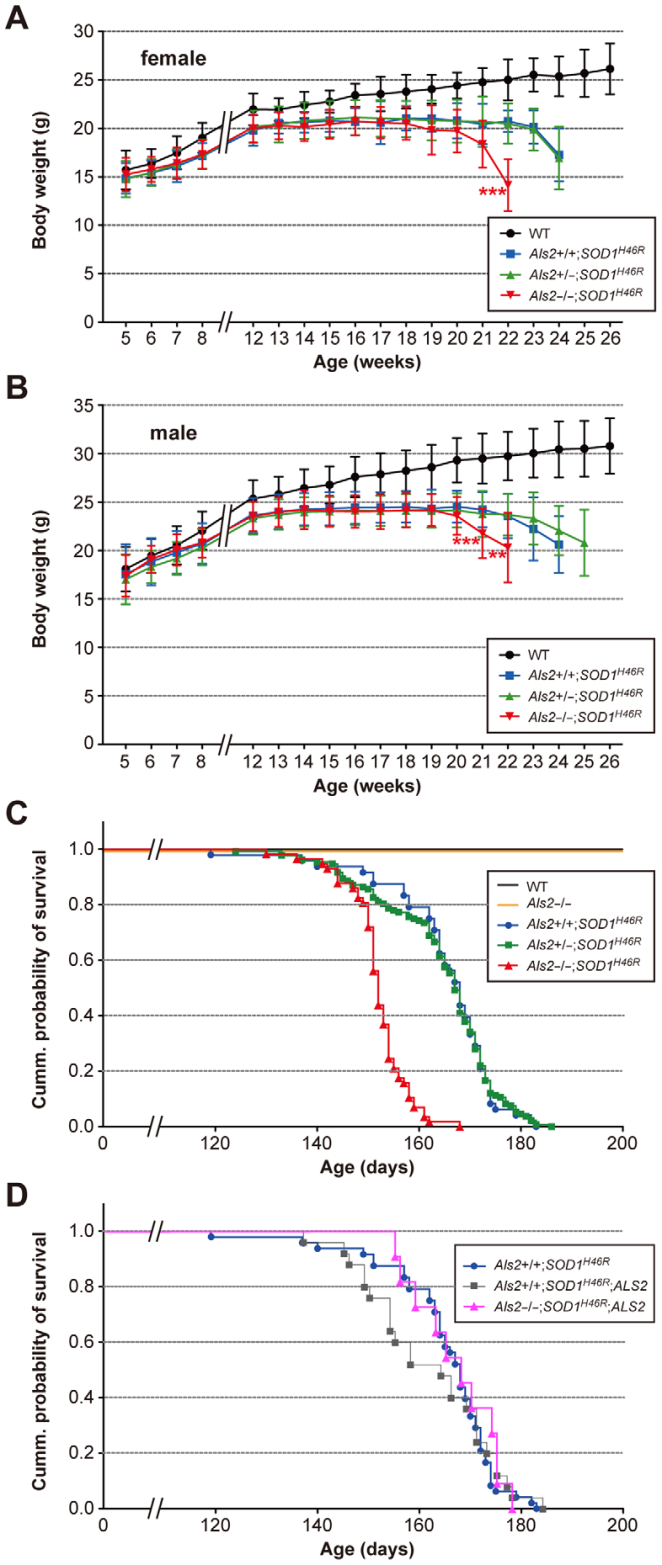
Loss of ALS2 results in accelerated body weight loss with shorter lifespan in *SOD1^H46R^* mice. (**A**) Growth curves for female mice [wild-type (WT) (black circle; n = 14–33), *Als2* ^+/+^;*SOD1^H46R^* (blue square; n = 6–30), *Als2* ^+/−^;*SOD1^H46R^* (green triangle; n = 28–84), and *Als2* ^−/−^;*SOD1^H46R^* (red inverted triangle; n = 7–44)], and (**B**) for male mice [WT (n = 41–48), *Als2* ^+/+^;*SOD1^H46R^* (n = 14–46), *Als2* ^+/−^;*SOD1^H46R^* (n = 9–76), and *Als2* ^−/−^;*SOD1^H46R^* (n = 8–36)]. (**A**–**B**) In either gender, age at which body weight loss began in *Als2* ^−/−^;*SOD1^H46R^* mice was earlier than that for *Als2* ^+/+^;*SOD1^H46R^* mice (female; ***p<0.001, at 22 weeks, male; ***p<0.001 and **p<0.01, at 21 and 22 weeks respectively). There were no differences in the mean values between *Als2* ^+/+^;*SOD1^H46R^* and *Als2* ^+/−^;*SOD1^H46R^* mice at any ages. The values for SOD1^H46R^-expressing mice (*Als2* ^+/+^;*SOD1^H46R^*, *Als2* ^+/−^;*SOD1^H46R^*, and *Als2* ^−/−^;*SOD1^H46R^*) later than 8 weeks of ages were all significantly lower than those for WT animals (levels of significance were not shown). Values are mean±SD. Statistical significance is evaluated by ANOVA with Tukey's *post hoc* test. (**C**) Survival curves for WT [black; n = 55 (female; n = 14, male; n = 41)], *Als2* ^−/−^ [orange; n = 78 (female; n = 32, male; n = 46)], *Als2* ^+/+^;*SOD1^H46R^* [blue circle; n = 48 (female; n = 13, male; n = 35)], *Als2* ^+/−^;*SOD1^H46R^* [green square; n = 132 (female; n = 63, male; n = 69)], and *Als2* ^−/−^;*SOD1^H46R^* [red triangle; n = 57 (female; n = 27, male; n = 30)]. Kaplan-Meier analysis identified significant difference between *Als2* ^−/−^;*SOD1^H46R^* and *Als2* ^+/+^;*SOD1^H46R^*, and between *Als2* ^−/−^;*SOD1^H46R^* and *Als2* ^+/−^;*SOD1^H46R^* (Log-rank test; *p*<0.0001). (**D**) Survival curves for *Als2* ^+/+^;*SOD1^H46R^* (blue circle; same as **C**), *Als2* ^+/+^;*SOD1^H46R^*;*ALS2* [gray square; n = 25 (female; n = 13, male; n = 12)], and *Als2* ^−/−^;*SOD1^H46R^*;*ALS2* [pink triangle; n = 11 (female; n = 4, male; n = 7)]. Kaplan-Meier analysis identified no significant differences between groups.

During the experimental periods, both wild-type and *Als2* ^−/−^ mice showed a constant increase in their body weight (Figure S2A and S2B), while mice carrying the *SOD1^H46R^* transgene reached their maximum body weight at 12–14 weeks of age, and terminally decreased as disease symptoms progressed (Figure 1A and 1B). Notably, loss of ALS2 in *SOD1^H46R^* mice (*Als2* ^−/−^;*SOD1^H46R^*) showed a marked and earlier decrease in their body weight compared to either *Als2* ^+/+^;*SOD1^H46R^* or *Als2* ^+/−^;*SOD1^H46R^* littermates (Figure 1A and 1B). Further, Kaplan-Meier survival analysis revealed that *Als2* ^−/−^;*SOD1^H46R^* mice died earlier than either *Als2* ^+/+^;*SOD1^H46R^* or *Als2* ^+/−^;*SOD1^H46R^* littermates (Figure 1C). Importantly, these exacerbated phenotypes by ALS2 loss were restorable by crossing to transgenic mice expressing human *ALS2* (*ALS2*-tg; line L34-1) (*Als2* ^−/−^;*SOD1^H46R^*;*ALS2* mice), albeit ALS2 overexpression *per se* did not show any obvious beneficial effects on lifespan in *SOD1^H46R^* mice (Figure 1D and S3). The results indicate that loss of ALS2 aggravates disease symptoms associated with SOD1^H46R^ expression in mice. By contrast, loss of ALS2 in *SOD1^G93A^* mice did not affect their lifespan (Figure S2C) as previously reported [29], [30], suggesting a limited role of ALS2 in SOD1^G93A^-mediated pathogenesis *in vivo*.

### Loss of ALS2 aggravates motor dysfunction in *SOD1^H46R^* mice

**Figure 2:**
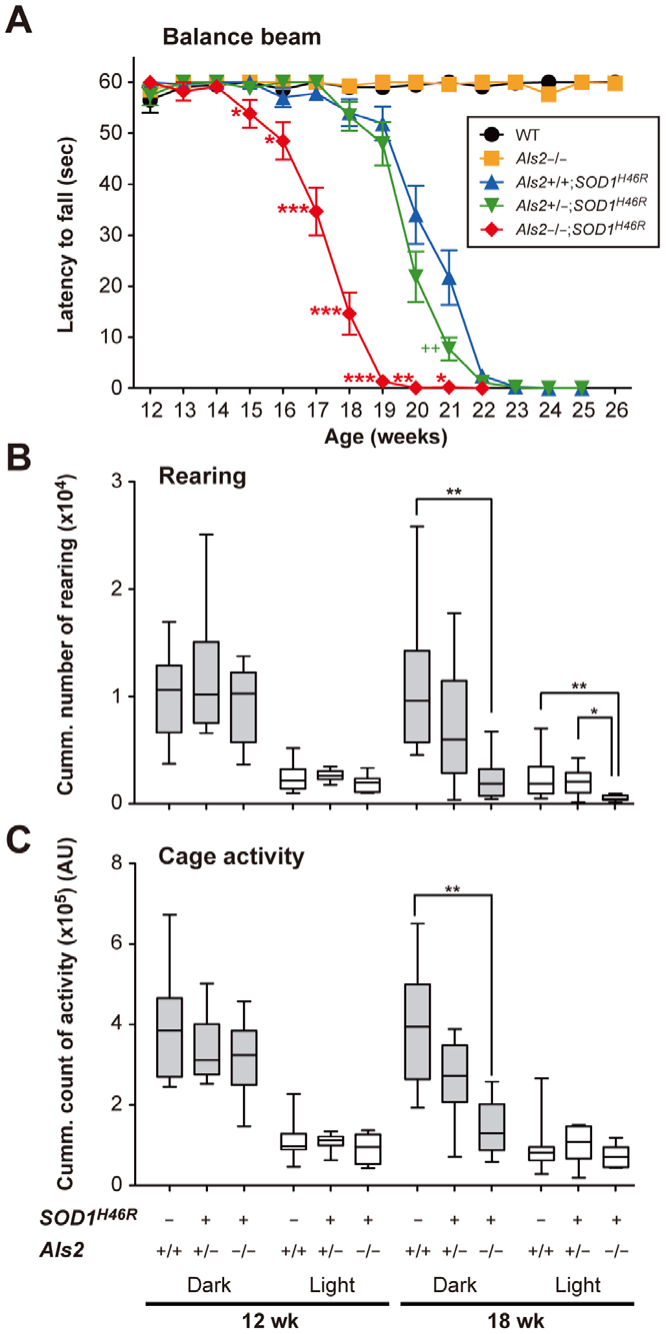
Loss of ALS2 aggravates motor dysfunction in *SOD1^H46R^* mice. (**A**) Changes in the balance beam test scores in wild-type (WT) (black circle), *Als2* ^−/−^ (orange square), *Als2* ^+/+^;*SOD1^H46R^* (blue triangle), *Als2* ^+/−^;*SOD1^H46R^* (green inverted triangle), and *Als2* ^−/−^;*SOD1^H46R^* (red diamond) mice. Values are means±SEM [each genotype; n = 20 (female; n = 10, male; n = 10)]. Statistical significance is evaluated by ANOVA with Scheffé's *post hoc* test. There are significant differences between *Als2* ^+/+^;*SOD1^H46R^* (blue) and *Als2* ^−/−^;*SOD1^H46R^* (red) mice (*p<0.05, **p<0.01, or ***p<0.001 at 15–21 weeks of ages), and between *Als2* ^+/+^;*SOD1^H46R^* (blue) and *Als2* ^+/−^;*SOD1^H46R^* (green) mice (++*p*<0.01 at 21 weeks of age). (**B**) The rearing and (**C**) cage activities in wild-type (*Als2* ^+/+^), *Als2* ^+/−^;*SOD1^H46R^*, and *Als2* ^−/−^;*SOD1^H46R^* mice in a dark (gray) and a light (white) cycle at 12 and 18 weeks of ages. Cumulative data counting for 7 consecutive days are shown as Box-Wisker plots [AU; arbitrary unit, each genotype; n = 8–10 (female)]. Statistical significance is evaluated by non-parametric ANOVA (Kruskal-Wallis) with Dunn's *post hoc* test. There are significant differences in the rearing activities (dark & light) between wild-type (*Als2* ^+/+^) and *Als2* ^−/−^;*SOD1^H46R^* mice (***p<0.01), in the rearing activities (light) between *Als2* ^+/−^;*SOD1^H46R^* and *Als2* ^−/−^;*SOD1^H46R^* mice (***p<0.05), and in the cage activities between wild-type (*Als2* ^+/+^) and *Als2* ^−/−^;*SOD1^H46R^* mice (***p<0.01) at 18 weeks of age.

As mice expressing SOD1^H46R^ exhibited progressive motor dysfunction and paralysis, particularly of the hind limbs, we next assessed whether loss of ALS2 in *SOD1^H46R^* mice affects the course of motor deficits by conducting quantitative behavioral analyses. First, to evaluate motor coordination and balance, we performed balance beam test, by which the onset of disease could be sensitively determined [34]. *Als2* ^−/−^;*SOD1^H46R^* mice showed an earlier motor dysfunction, in which the onset of disease (∼15 weeks of age) was approximately 3 weeks earlier than those (∼18 weeks of age) of *Als2* ^+/+^;*SOD1^H46R^* or *Als2* ^+/−^;*SOD1^H46R^* littermates (Figure 2A). Further, analyses of rearing and cage activities revealed that although there were no significant differences in their activities among all pre-symptomatic mice (12 weeks of age) with different genotypes, *Als2* ^−/−^;*SOD1^H46R^* mice showed a significantly lower spontaneous motor activity than wild-type or *Als2* ^+/−^;*SOD1^H46R^* littermates at a later stage of the disease (18 week of age) (Figure 2B and 2C).

### *SOD1^H46R^* mice show an axonal degeneration in the spinal tracts from an early symptomatic stage

**Figure 3:**
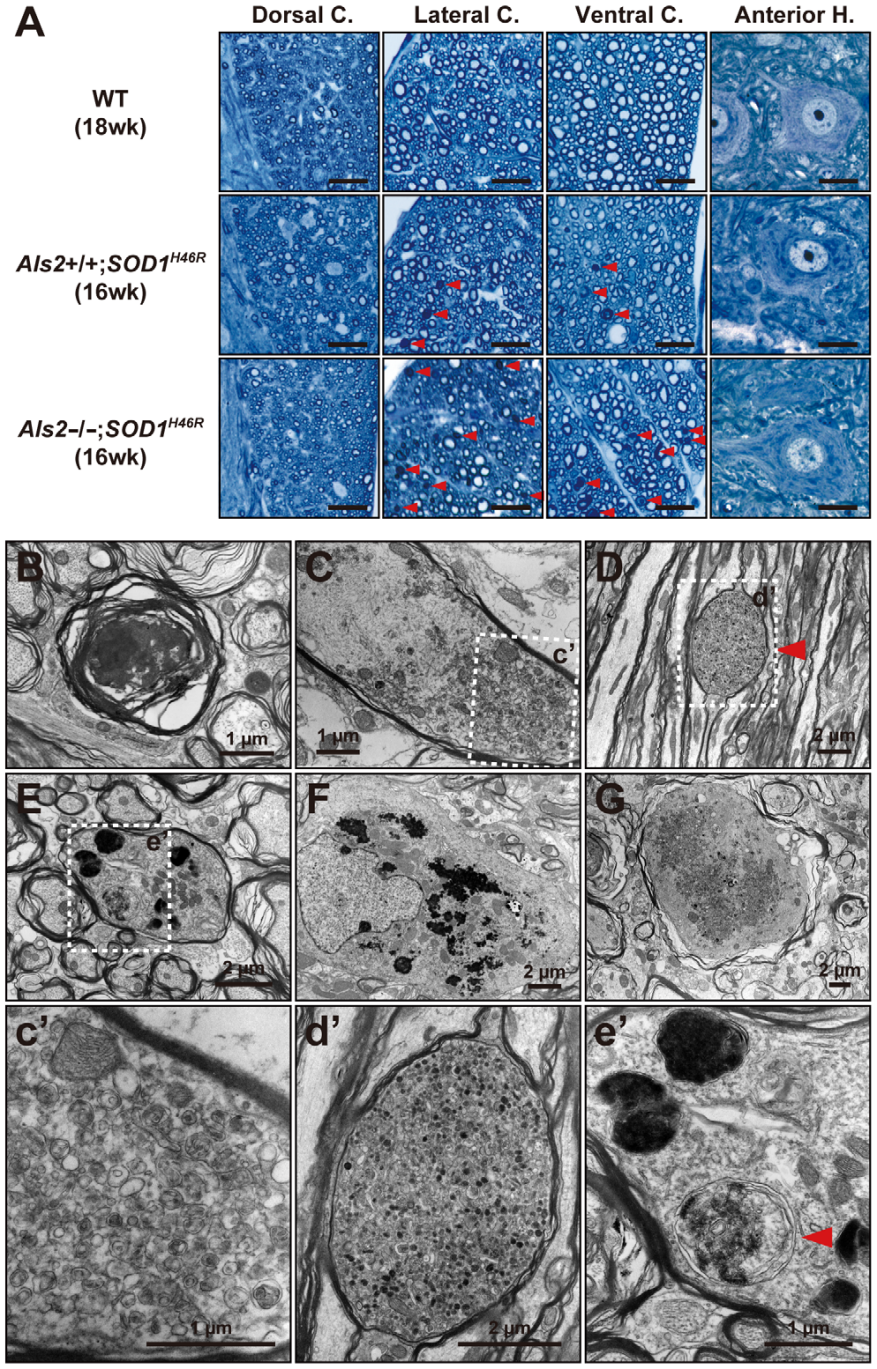
SOD1^H46R^-expressing mice show axonal degeneration and swelling in the spinal cord. (**A**) Representative toluidine blue staining of the transverse section of lumbar spinal cord (L4–L5) from 18-week-old wild-type (WT; upper row), 16-week-old *Als2* ^+/+^;*SOD1^H46R^* (middle row), and 16-week-old *Als2* ^−/−^;*SOD1^H46R^* (lower row) mice. Images for the dorsal columns, lateral columns, ventral columns, and ventral horn cells were shown. Red arrowheads indicate degenerating axons. Axonal degeneration is most prominent in *Als2* ^−/−^;*SOD1^H46R^* mice. Scale bars = 20 µm. (**B**–**F**) Representative electron micrographs of lumbar (L4–L5) spinal axons from *Als2* ^−/−^;*SOD1^H46R^* mouse at 16 weeks (**B**–**F**) and 8 weeks (**G**). Degenerating axon (**B**), axon accumulating fibrillar materials and multivesicular bodies (**C** and **c′**), membrane saccule containing granular/osmiophilic aggregates and autophagosome-like vesicles (**D** and **d′**, red arrowhead), axon containing osmiophilic and autophagosome-like (red arrowhead) vesicles (**E** and **e′**), astrocyte containing osmiophilic aggregates (**F**), and swollen axon accumulating granular aggregates and vesicles (**G**), are shown. Scale bars are as indicated.

To determine whether these motor phenotypes are associated with motor neuron degeneration, we conducted histological analyses using early symptomatic mice (16–18 weeks of age). Although there were no evidences for the motor neuron loss at this stage, a wide-spread axonal degeneration in the spinal tracts of the lateral and ventral columns was evident, particularly in *Als2* ^−/−^;*SOD1^H46R^* mice (Figure 3A).

### Osmiophilic granular aggregates, multivesicular bodies, and autophagosome-like structures are accumulated in the spinal cord of *SOD1^H46R^* mice

To investigate the histopathology in more detail, we next conducted an electron microscopic (EM) analysis. Although there were no observable abnormalities in soma of motor neurons (data not shown), we observed the degenerative and swollen axons with the accumulation of granular aggregates, disorganized fibrillar materials, multivesicular bodies (MVBs), and/or autophagosome-like vesicles in the spinal cord of *Als2* ^−/−^;*SOD1^H46R^* and *Als2* ^+/+^;*SOD1^H46R^* mice at an early symptomatic stage (Figure 3B and 3C). Further, membrane saccules containing granular/osmiophilic aggregates (Figure 3D) and autophagosome-like vesicles (Figure 3E) were observed. Astrocytes containing osmiophillic aggregates were also occasionally observed (Figure 3F). Notably, in *Als2* ^−/−^;*SOD1^H46R^* mice, these pathologic phenotypes were evident even at 8 weeks of pre-symptomatic stage (Figure 3G), but not in a same stage of *Als2* ^+/+^;*SOD1^H46R^* mice (data not shown). These data underscore the relevance of axonal dysfunction and/or degeneration, probably caused by the dysfunctional axonal trafficking, on the disease onset in SOD1^H46R^-expressing mice.

### Loss of ALS2 promotes the progressive accumulation of insoluble proteins in the spinal cord of *SOD1^H46R^* mice

**Figure 4:**
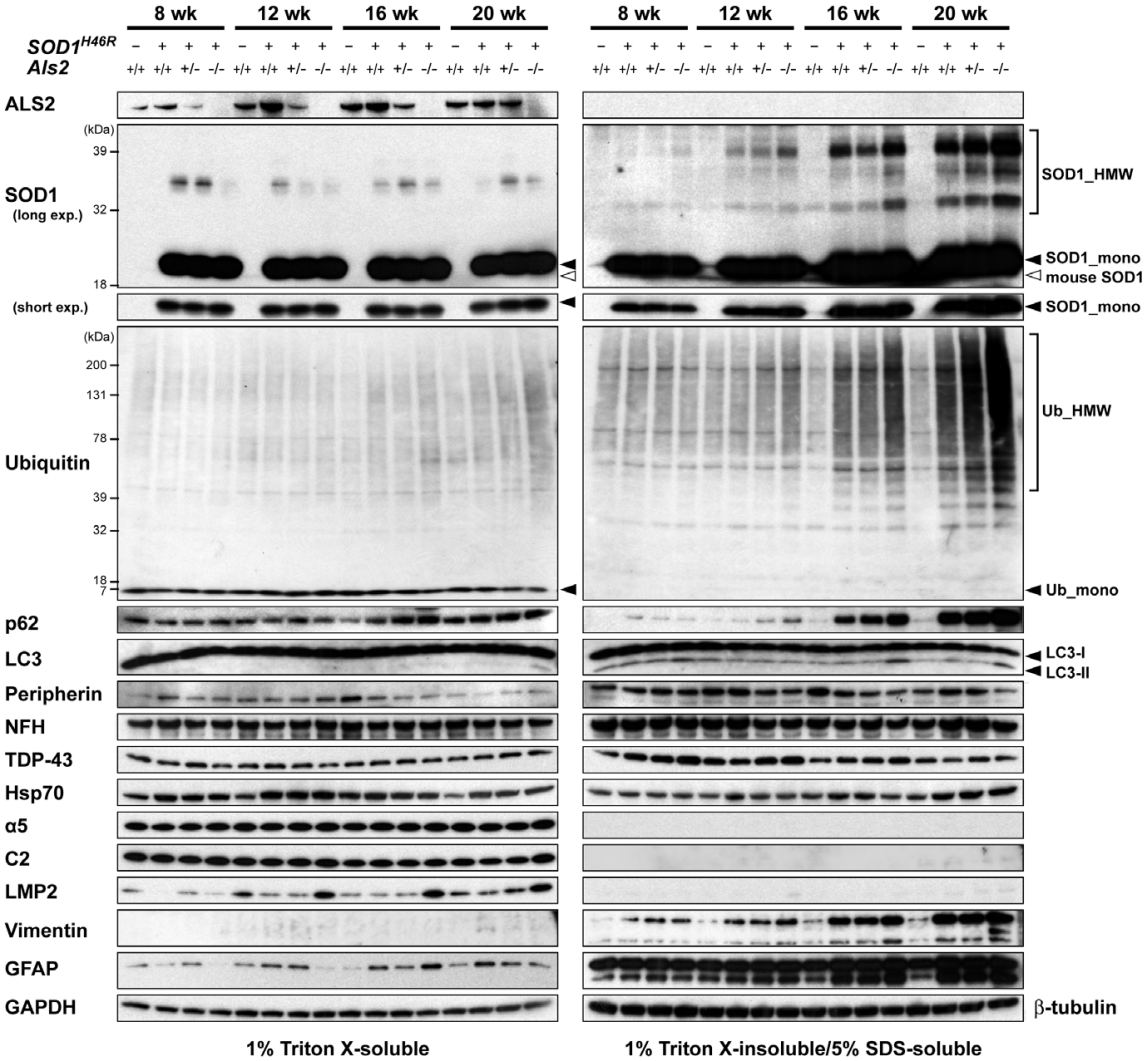
ALS2 loss promotes an accumulation of insoluble proteins in the spinal cord of *SOD1^H46R^* mice. Western blot analysis of the levels of proteins, including ALS2, SOD1, ubiquitin (Ub), polyubiquitin binding protein p62/SQSTM1 (p62), microtubule-associated protein 1-light chain 3 (LC3), peripherin, neurofilament heavy chain (NFH), TAR DNA-binding protein 43-kD (TDP-43), heat-shock protein Hsp70, 20S proteasome subunits (α5, C2, and LMP2), vimentin, and glial fibrillary acidic protein (GFAP), in the lumbo-sacral cord from 8, 12, 16, and 20 week-old mice with four distinct genotypes; wild-type (*Als2* ^+/+^), *Als2* ^+/+^;*SOD1^H46R^*, *Als2* ^+/−^;*SOD1^H46R^*, and *Als2* ^−/−^;*SOD1^H46R^*. Two fractions; 1% Triton X-soluble fraction (TX-soluble; left panels) and 1% Triton X-insoluble/5% SDS-soluble fraction (TX-insoluble; right panels) were analyzed. SOD1_mono and SOD1_HMW represent monomeric and high molecular-weight (aggregated) forms of SOD1, respectively. Ub_mono and Ub_HMW represent monomeric ubiquitin and the polyubiquitinated proteins, respectively. LC-I and LC-II are cytosolic and lipidated forms of LC3, respectively. Glyceraldehyde 3-phosphate dehydrogenase (GAPDH) and β-tubulin served as controls for TX-soluble and TX-insoluble fractions, respectively.

First, we confirmed that there were no significant differences in the expression levels of the *SOD1^H46R^* transcript (Figure S4A) and soluble SOD1^H46R^ protein (Figure 4, S5A, and S6A) among three different groups of mice carrying the human *SOD1* transgene; i.e., *Als2* ^+/+^;*SOD1^H46R^*, *Als2* ^+/−^;*SOD1^H46R^*, and *Als2* ^−/−^;*SOD1^H46R^*. These results indicate that the exacerbation of motor dysfunction seen in *SOD1^H46R^* mice lacking ALS2 was not simply due to the increased level of the SOD1^H46R^ expression.

To identify the molecular factors that were associated with behavioral and pathological features observed in *Als2* ^−/−^;*SOD1^H46R^* mice, we conducted western blot analysis of the lumbo-sacral cord extracts using a panel of antibodies, and performed their quantitative analyses. Although the levels of the ALS-related neuronal intermediate filament proteins [peripherin and neurofilament heavy chain (NFH)] [35] and the ALS-causative gene product [TAR DNA-binding protein 43-kD (TDP-43)] [1], [2] were unchanged, a progressive accumulation of insoluble high-molecular weight (HMW) SOD1 and poly-ubiquitinated proteins was observed in mice expressing SOD1^H46R^, particularly in those lacking ALS2 (*Als2* ^−/−^;*SOD1^H46R^*) (Figure 4, S5B, S5C, S5E, S6A, and S6B). Further, from 16 weeks of pre- and early-symptomatic stage, glial intermediate filament proteins, vimentin and glial fibrillary acidic protein (GFAP), were accumulated (Figure 4, S5J, S5K, and S6E). Remarkably, the levels of two macroautophagy (hereafter referred to as autophagy)-associated proteins, polyubiquitin binding protein p62/SQSTM1 (p62) and a lipidated-form of microtubule-associated protein 1-light chain 3 (LC3-II), both of which were selectively degraded by autophagy-lysosomal system [36], were significantly increased in insoluble fractions of *Als2* ^−/−^;*SOD1^H46R^* mice (Figure 4, S5G, S5I, S6C, and S6D). Similar results were obtained in samples from the brainstem, cerebellum, and cervical cord, but not from the cerebral cortex (Figure S7). A quantitative reverse transcriptase PCR (qRT-PCR) revealed that although expression of *Vim* and *Gfap* was significantly upregulated as the disease progressed, no transcriptional activation of *SOD1^H46R^*, *Sqstm1* (p62), and *Map1lc3a*/*Map1lc3b* (LC3A/LC3B) was observed (Figure S4). These results suggest that the accumulation of insoluble mutant SOD1, p62, and LC3-II is not simply due to an increased expression of these proteins, but rather to a decrease in their degradation. Since mutant SOD1 is degraded by both the proteasome and autophagy [37], [38], impairment of the ubiquitin-proteasome system (UPS) and/or the autophagy-endolysosomal system could result in the accumulation of such insoluble proteins in *SOD1^H46R^* mice, particularly in those lacking ALS2.

### Proteasome activity in the spinal cord is increased as the disease progresses

**Figure 5:**
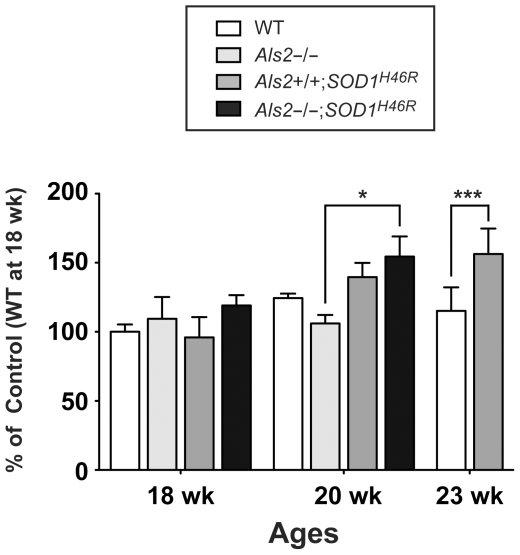
Proteasome activity in the spinal cord of *SOD1^H46R^* mice is increased as the disease progresses. Chymotrypsin-like activity in the lumbo-sacral cord from 18, 20, and 23 week-old mice with four distinct genotypes; wild-type (WT), *Als2* ^−/−^, *Als2* ^+/+^;*SOD1^H46R^*, and *Als2* ^−/−^;*SOD1^H46R^* are measured. Values are mean ± SD (n = 3–5) in percent (%) relative to 18 week-old wild-type mice. Statistical significance is evaluated by ANOVA with Bonferroni's *post hoc* test (*p<0.05, ***p<0.001).

To investigate the contribution of UPS impairment on the protein accumulation observed, we analyzed the catalytic activity for the 20S proteasome in the spinal cord from mice with different genotypes. Surprisingly, the proteasomal activity was induced rather than impaired in SOD1^H46R^-expressing mice at a late symptomatic stage (20 and 23 weeks of age) (Figure 5). Although loss of ALS2 by itself did not affect the proteasome activity (wild-type vs *Als2* ^−/−^ at 18 weeks of age), ALS2 deficiency in *SOD1^H46R^* mice seems to result in the earlier enhancement of proteasome activity in the spinal cord (20 weeks of age) (Figure 5), which might be correlated with the disease progression. Importantly, the levels of LMP2, a inducible subunit of immunoproteasome, was increased in symptomatic *Als2* ^−/−^;*SOD1^H46R^* mice (Figure 4), consistent with recent findings [39], [40]. Since the levels of the constitutive subunits of 20S proteasome (α5 and C2) and one of the molecular chaperone Hsp70 were unchanged (Figure 4), the UPS was not severely affected in *SOD1^H46R^* mice, at least, at the disease stages examined, but rather enhanced in certain cell types within the spinal cord in response to the disease progression. Thus, the accelerated accumulation of HMW SOD1 and polyubiquitinated proteins from an early symptomatic stage in *SOD1^H46R^* mice is not simply due to impairment of the UPS.

### *SOD1^H46R^* mice reveal the accumulation of ubiquitin, p62, and LC3 in the spinal cord

To investigate the cellular localization of the accumulated insoluble proteins in the spinal cord, we performed immunohistochemical analysis. In mice expressing SOD1^H46R^, a higher SOD1-immunoreactivity was observed throughout white and gray matters of the spinal cord (Figure S8A). However, unlike in a case of *SOD1^G93A^* mice [41], no obvious SOD1-positive inclusions were detected, at least, in the samples from early symptomatic mice (Figure S8B), consistent with the notion that SOD1^H46R^ shows minimal tendency to form the aggregates or inclusions when compared with other aggregation-prone SOD1 mutants, such as SOD1^G93A^ [42]. HMW insoluble SOD1 observed in immunoblots (Figure 4) may represent oligomeric forms of SOD1^H46R^ that are not analogous to the aggregates of other SOD1 mutants, and are thus invisible under the light-microscopic observations.

**Figure 6:**
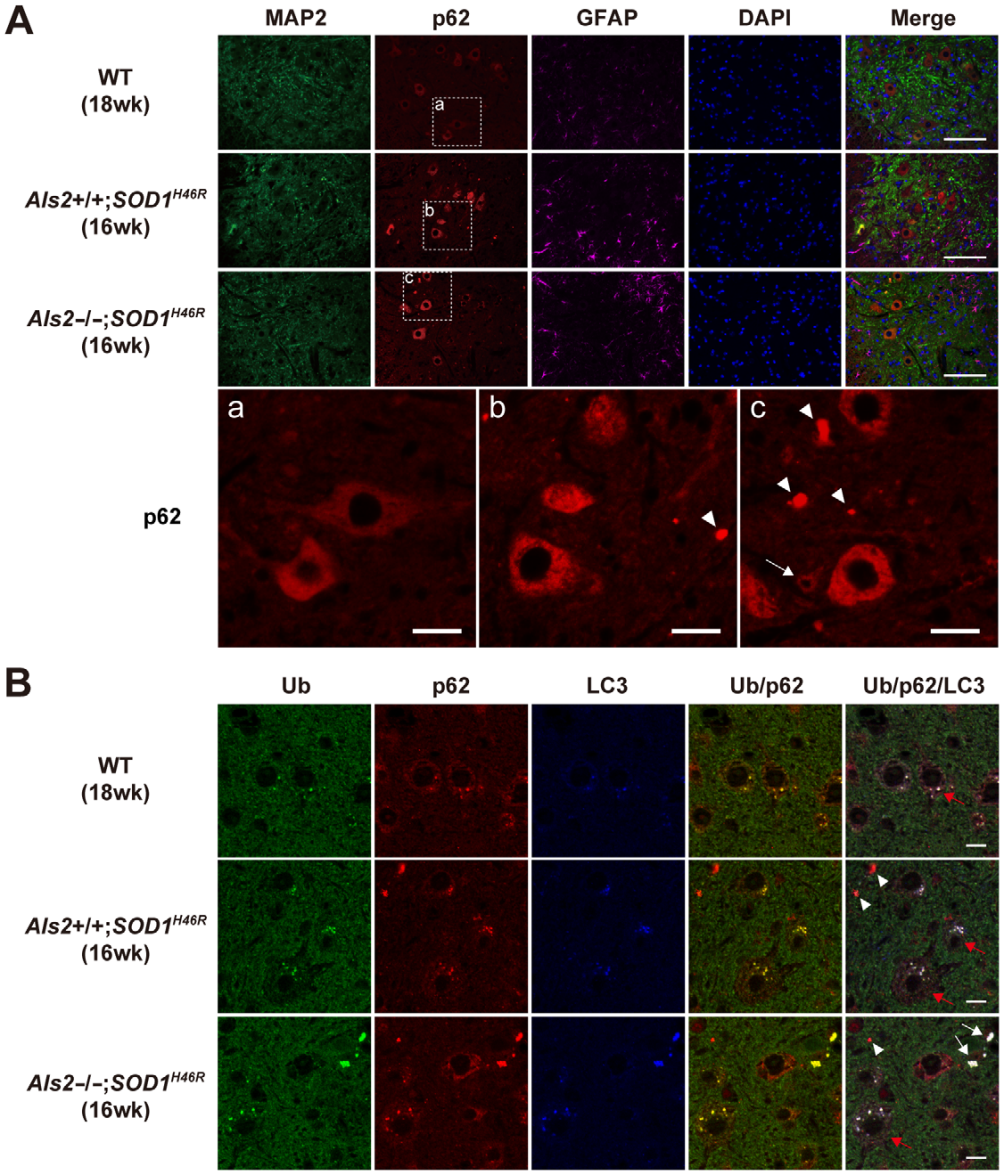
SOD1^H46R^-expressing mice show the accumulation of ubiquitin, p62, and LC3 in the spinal cord. (**A**) Representative images of triple immunostaining with MAP2 (green), p62 (red), and GFAP (pink) for the ventral horn of the lumbar spinal cord (L4–L5) from 18-week-old wild-type (WT; 1^st^ row), 16-week-old *Als2* ^+/+^;*SOD1^H46R^* (2^nd^ row), and 16-week-old *Als2* ^−/−^;*SOD1^H46R^* (3^rd^ row) mice. The nuclei were counterstained with DAPI (Blue). Scale bars = 100 µm. (Lower row) Higher magnification images of p62 immunostaining for the ventral horn of the spinal cord from (**a**) 18-week-old WT, (**b**) 16-week-old *Als2* ^+/+^;*SOD1^H46R^*, and (**c**) 16-week-old *Als2* ^−/−^;*SOD1^H46R^* mice. It is notable that loss of ALS2 results in an increased number of p62-positive smaller-sized neurons (white arrow) and p62-positive extracellular aggregates (white arrowheads). Scale bars = 20 µm. (**B**) Representative images of triple immunostaining with ubiquitin (Ub) (green), p62 (red), and LC3 (blue) for the ventral horn of the lumbar spinal cord (L4–L5) from 18-week-old WT (upper row), 16-week-old *Als2* ^+/+^;*SOD1^H46R^* (middle row), and 16-week-old *Als2* ^−/−^;*SOD1^H46R^* (lower row) mice. Red and white arrows represent large motor neurons containing cytoplasmic Ub/p62/LC3-positive puncta and extracellular Ub/p62/LC3-positive aggregates, respectively. The p62-single positive aggregates are also observed (white arrowheads). Scale bars = 10 µm.

Expression of p62 was evident in microtubule-associated protein 2 (MAP2)-positive large motor neurons but not in GFAP-positive astrocytes in the spinal cord, and the levels of p62 were higher in mutant SOD1-expressing mice (*Als2* ^+/+^;*SOD1^H46R^* and *Als2* ^−/−^;*SOD1^H46R^*) than in wild-type control (Figure 6A). Further, compared to *Als2* ^+/+^;*SOD1^H46R^* mice, *Als2* ^−/−^;*SOD1^H46R^* mice showed more intense p62-immunoreactivities in both large and smaller-sized neurons, and the formation of large p62-positive aggregates was more prominent (Figure 6A). Co-immunostaining of p62 with a number of cellular markers revealed that although the p62-positive large aggregates were occasionally colocalized with MAP2, they were mostly surrounded by astrocytes, but not colocalized with astrocyte, myelin, oligodendrocyte, or microglia markers (Figure 6 and data not shown). These results suggest that the large aggregates are present in extracellular spaces. Importantly, triple-immunostaning demonstrated that these large aggregates simultaneously contained p62, ubiquitin, and LC3 (Figure 6B). Thus, it is possible that these ubiquitin/p62/LC3-positive aggregates in the extracellular space are derived from the degenerated dendrites and/or axons. Since the accumulated polyubiquitinated proteins, p62, and LC3-II are a hallmark of the autophagic defect [36], we hypothesize that loss of ALS2 associates with deregulation of the endolysosomal bulk protein degradation, thereby aggravating the SOD1^H46R^-associated disease symptoms in mice.

### ALS2 is colocalized with autophagosomal proteins in cultured cells

**Figure 7:**
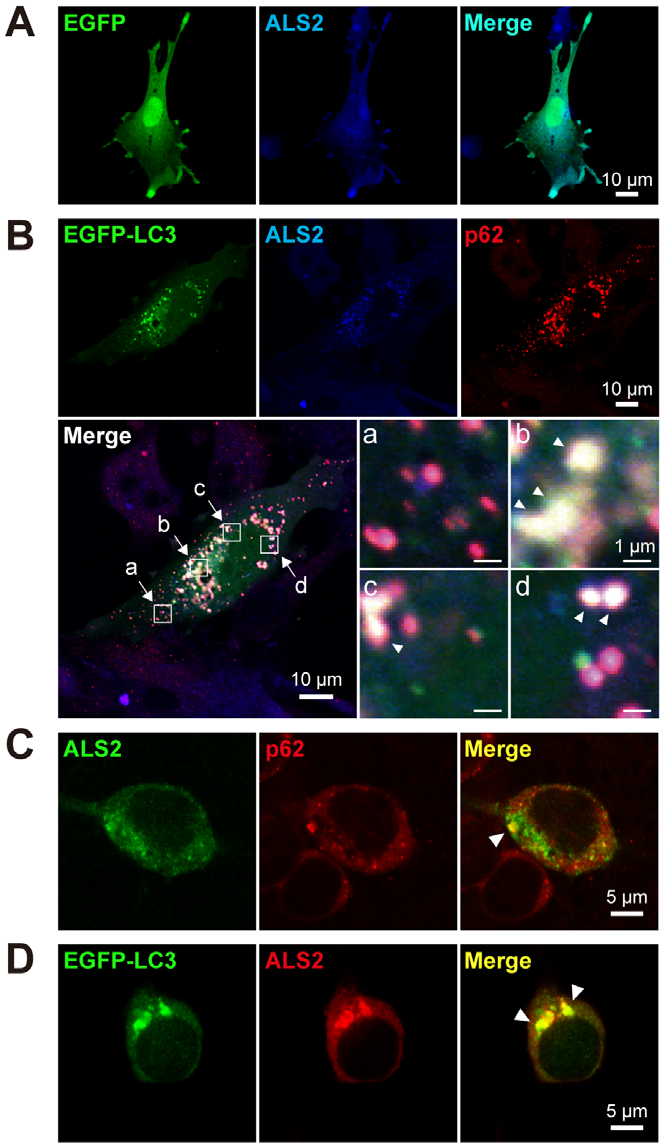
ALS2 is colocalized with LC3 and p62 onto vesicular compartments in cultured cells. (**A**) Diffused cytoplasmic distribution of ectopically expressed ALS2 in wild-type fibroblasts. The cells were cotransfected with EGFP (green) and ALS2 (blue). Right column displays the merged image. (**B**) Punctate distribution of ectopically expressed ALS2 (blue) colocalizing with EGFP-LC3 (green) and endogenous p62 (red) in wild-type fibroblasts. The cells were cotransfected with EGFP-LC3 and ALS2. An ectopic expression of EGFP-LC3 enhances the vesicular localization of ALS2 in fibroblasts when compared with EGFP (**A**). Composite images representing ALS2/p62-double-positive (**a**) and LC3/ALS2/p62-triple-positive puncta and/or vesicles (**b**, **c**, and **d**; white arrowheads) at a higher magnification are shown. (**C**) Ectopically expressed ALS2 (green) is colocalized with endogenous p62 (red) in mice primary hippocampal neurons (white arrowhead). The cells (DIV11; early stage 5) were transfected with ALS2, followed by a 12 hr of starvation. (**D**) Ectopically expressed EGFP-LC3 (green) and ALS2 (red) are colocalized onto perinuclear puncta/vesicles in mice primary hippocampal neurons (white arrowheads). The cells (DIV11; early stage 5) were cotransfected with EGFP-LC3 and ALS2, followed by a 12 hr of starvation. (**A**–**D**) Scale bars are as indicated.

To investigate the physiological role of ALS2 and its relationship with the autophagy-associated endolysosomal system, we performed a colocalization study for ALS2, p62, LC3, and other organelle markers using cultured cells. Distribution of ectopically expressed ALS2 and EGFP-LC3 extensively overlapped with endogenous p62 in vesicular compartments in fibroblasts (Figure 7A and 7B). Further, ectopically expressed ALS2 was colocalized with either endogenous p62 (Figure 7C) or EGFP-LC3 (Figure 7D) onto the vesicular compartments in primary hippocampal neurons. Analysis with organella markers in HeLa cells revealed that ALS2/LC3 double-positive vesicles were partially co-stained with either p62, early endosome antigen 1 (EEA1; early endosome marker), or lysosome-associated membrane protein 2 (LAMP2; late endosome/lysosome marker) (Figure S9), but not with the mitochondrial, ER, and Golgi markers (data not shown). These results indicate that ALS2 is present not only onto endosomes and macropinosomes [15], [16], but also onto autophagosomes and/or autophagosome/endosome hybrid vesicular compartments called amphisomes [43].

### Loss of ALS2 results in a decreased level of lysosomal clearance of autophagy-associated proteins in fibroblasts

**Figure 8:**
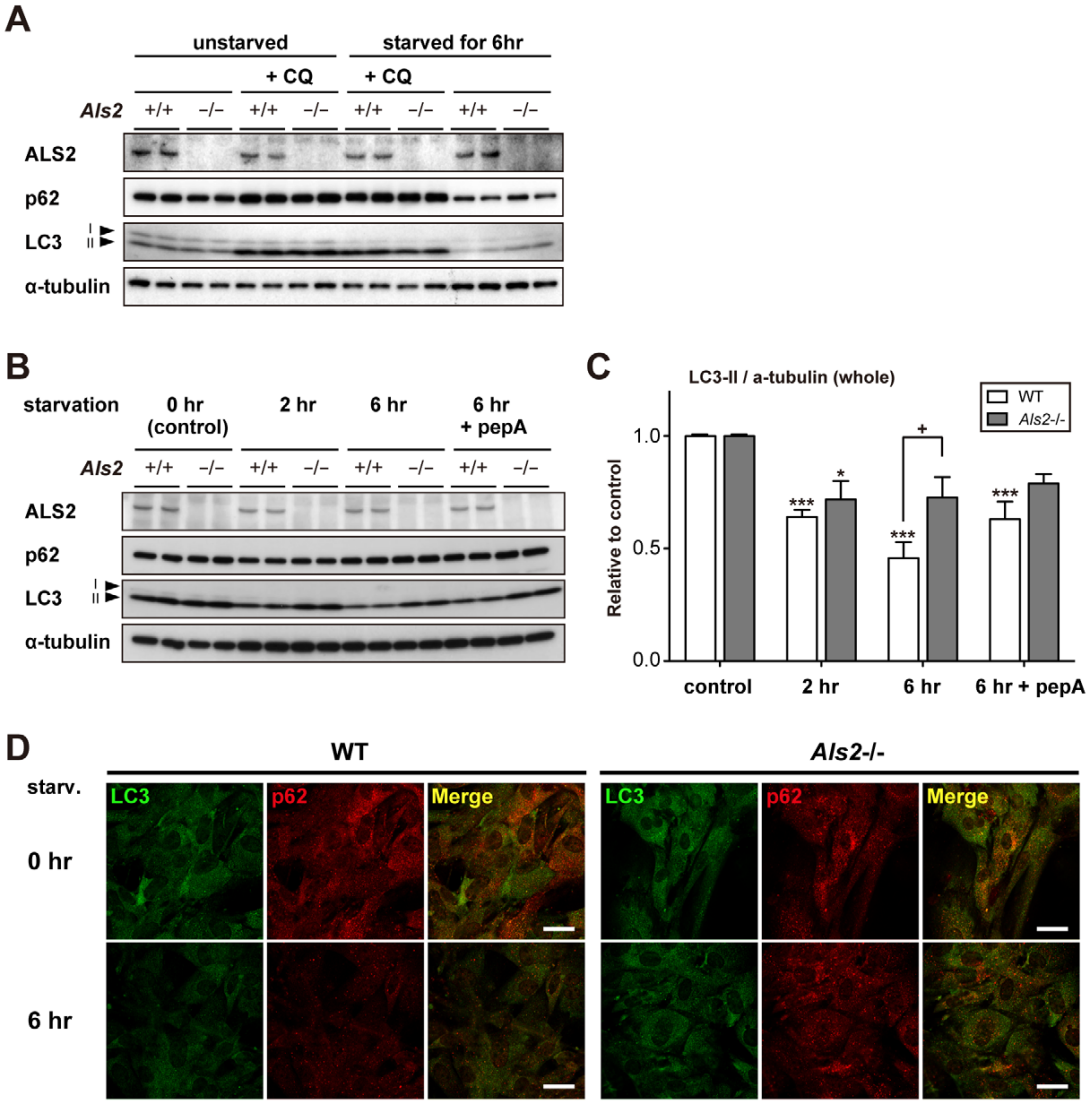
Loss of ALS2 results in decreased levels of the lysosome-dependent degradation of LC3 in fibroblasts. (**A**) Effect of ALS2 expression on the autophagic flux in fibroblasts. Fibroblasts derived from either wild-type (*Als2* ^+/+^) or *Als2* ^−/−^ mice were incubated in a starvation medium with or without 0.5 µM chloroquine (CQ) for 6 hr. Equal amount of protein from total lysates were analyzed by immunoblotting using antibodies as indicated. Alpha(α)-tubulin served as control. (**B**) Effect of ALS2 expression on the autophagic flux in fibroblasts. Fibroblasts derived from either wild-type (*Als2* ^+/+^) or *Als2* ^−/−^ mice were incubated in a starvation medium with or without 20 µg/ml pepstatin A (pepA) for indicated periods. Equal amount of protein from total lysates were analyzed by immunoblotting using antibodies as indicated. Alpha(α)-tubulin served as control. (**C**) Quantitative densitometry for the levels of LC3-II immunoreactive signals shown in **B**. Data were normalized by the levels of α-tubulin (LC3-II/α-tubulin). Values are mean±SEM (n = 4) in an arbitrary unit relative to control. Statistical significance is evaluated by ANOVA with Bonferroni's *post hoc* test (one-way, compared with respective controls; *p<0.05, ***p<0.001, and two-way, compared between WT and *Als2* ^−/−^; +*p*<0.05). (**D**) Representative images for double immunostaining with LC3 (green) and p62 (red) in fibroblasts. Fibroblasts from wild-type (WT) and *Als2* ^−/−^ mice were either left unstarved (0 hr) (upper) or starved for 6 hr (lower). It is notable that a 6 hr of starvation leads to decreased levels of the LC3- and p62-immunoreactive signals in WT cells, but not in *Als2* ^−/−^ cells. Scale bars = 50 µm.

Previously, it has been shown that ALS2 regulates the trafficking and clearance of internalized molecules such as epidermal growth factor (EGF) [20] and glutamate receptors [44] in cultured cells. To further clarify the functional interaction of ALS2 with the autophagosomal-endolysosomal protein degradation in general, fibroblasts derived from wild-type and *Als2* ^−/−^ mice were subjected to autophagic-flux analysis [45]. Since the intracellular levels of LC3-II correlate with the number of autophagosomes, which is regulated by a balance between autophagosome formation and degradation, the LC3-II levels in the presence or absence of lysosomal inhibitors can be used as “autophagomometer” to measure the autophagic-flux [45]. Under steady-state conditions (unstarved), the treatment with chloroquine (CQ), a lysosomotropic agent that inhibits the lysosomal proteases, resulted in a comparable increase in the LC3-II level in *Als2* ^−/−^ cells with those in wild-type (Figure 8A), indicating that loss of ALS2 by itself does not seem to affect the formation of autophagosomes in cells. Further, a short-term starvation in wild-type and *Als2* ^−/−^ fibroblasts resulted in decreased levels of p62 and LC3-II, indicating an enhancement of autophagy-dependent protein degradation in both cell types (Figure 8A). Notably, under such starved conditions, the LC3-II levels in *Als2* ^−/−^ cells were significantly higher than those in wild-type cells (Figure 8A), suggesting that a degree of autophagic clearance was affected by ALS2 loss.

To confirm this, a quantitative analysis of the LC3-II levels with a different inhibitor (pepstatin A) was performed. Wild-type fibroblasts showed a progressive decrease in the levels of LC3-II, which was partially restored by the pepstatin treatment. By contrast, *Als2* ^−/−^ cells exhibited the least effects after a similar starvation, in which the LC3-II levels in *Als2* ^−/−^ cells was significantly higher than those in wild-type *Als2* ^−/−^ cells, and stayed unchanged under the presence of pepstatin (Figure 8B and 8C), indicating an inefficient lysosomal clearance of LC3-II in *Als2* ^−/−^ cells. Double-immunostaining experiments also confirmed that loss of ALS2 led to sustained fluorescent signals of p62 and LC3 after starvation in fibroblasts (Figure 8D). Importantly, fibroblasts derived from ALS2 overexpressing mice (*ALS2*-tg line L6-2) (Figure S3) showed the opposite effects in which the clearance of LC3-II was accelerated (Figure S10).

We further performed a small interfering RNA (siRNA)-mediated *ALS2* knockdown in HeLa cells, and revealed that the suppression of ALS2 markedly increased a steady-state level of LC3-II (Figure S11A and S11B) with accompanying large perinuclear aggregates and/or vesicles containing LC3 (Figure S11D). Although the apparent level of p62 was unchanged, p62 was also re-distributed to the perinuclear LC3-positive compartments by suppressing the *ALS2* expression (Figure S11C and S11D). Again, autophagic-flux analysis with the CQ treatment showed that *ALS2* knockdown by itself did not induce autophagy in HeLa cells (Figure S11C).

Altogether, it is suggested that the accumulation of LC3-II observed in ALS2 deficient cells is due to a decreased level of clearance, rather than an increased formation, of autophagosomes and/or amphisomes. Thus, ALS2 might play an active role in the autophagosomal-endolysosomal trafficking in fibroblasts and HeLa cells.

### ALS2 regulates the autophagosomal-endolysosomal trafficking in cultured spinal motor neurons

**Figure 9:**
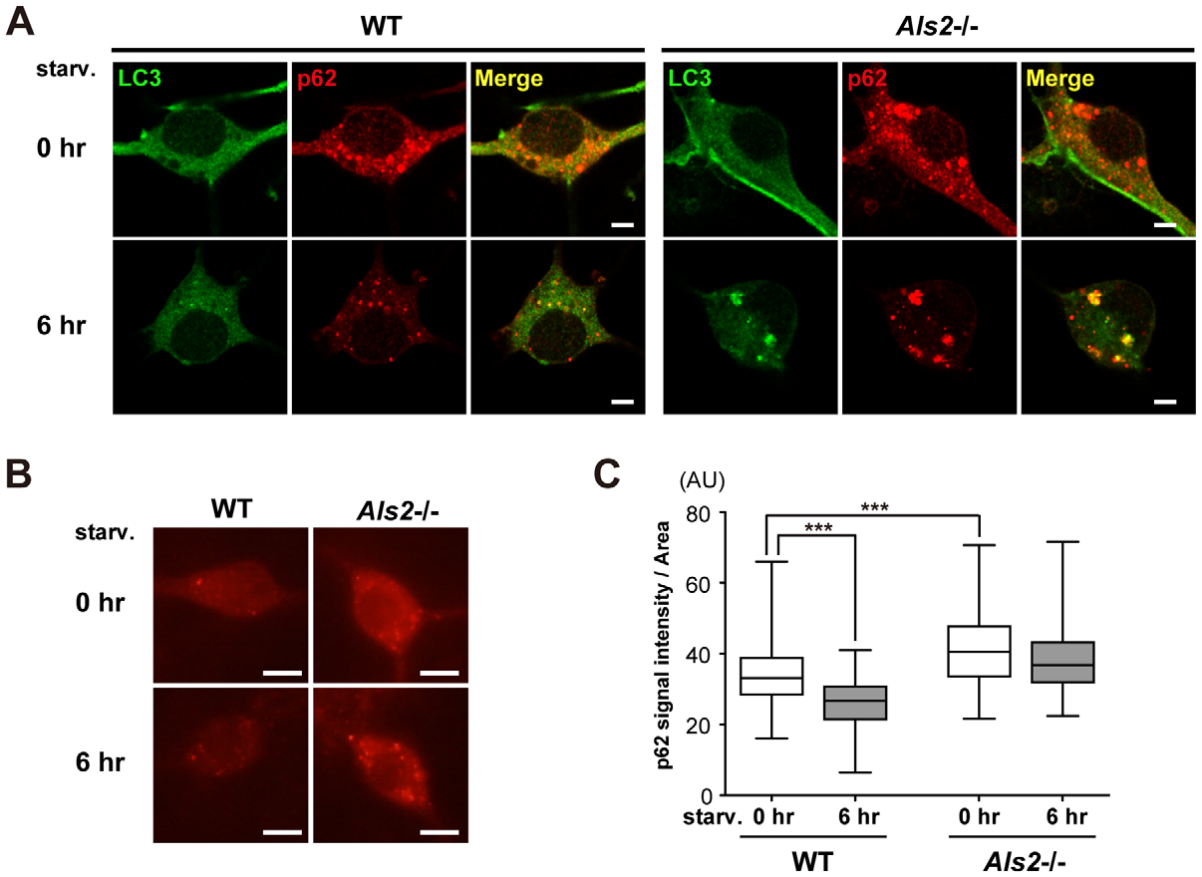
Loss of ALS2 lowers the starvation-induced clearance of p62 in cultured motor neurons. (**A**) Representative confocal images for double immunostaining with LC3 (green) and p62 (red) in primary spinal motor neurons. The cells (DIV14) from wild-type (WT) and *Als2* ^−/−^ mice were either left unstarved (0 hr) (upper) or starved for 6 hr (lower). A 6 hr of starvation leads to decreased levels of the LC3- and p62-immunoreactive signals in WT cells. It is notable that a same treatment to *Als2* ^−/−^ cells results in the enlargement of LC3/p62-double positive puncta/vesicles in soma. Scale bars = 5 µm. (**B**) Representative fluorescent microscopic images used for the quantitative analysis of p62-immunostaining in primary spinal motor neurons shown in **C**. The cells (DIV14) derived from wild-type (WT) and *Als2* ^−/−^ mice were either left unstarved (0 hr) or starved for 6 hr. Scale bars = 10 µm. (**C**) Quantitation of the p62-immunoreactivity in randomly selected spinal neurons. Signal intensities for the p62-immunoreactivity relative to unit area of soma (pixel) (AU; arbitrary unit) are shown as Box-Wisker plots [wild-type (WT); 0 hr (n = 149) and 6 hr (n = 123), *Als2* ^−/−^; 0 hr (n = 127) and 6 hr (n = 139)]. Statistical significance is evaluated by non-parametric ANOVA (Kruskal-Wallis) followed by Dunn's *post hoc* test. There is a significant difference in the basal levels of p62 immunoreactive intensities between WT and *Als2* ^−/−^ cells (***p<0.001). A 6 hr of starvation results in a significant decrease in the signal intensities in WT [compared 0 hr (white) with 6 hr (gray), ***p<0.001], but not in *Als2* ^−/−^ cells.

To determine whether ALS2 acts as a modulator for the endolysosomal system in neuronal cells, we investigated the changes in the level and distribution of p62 and LC3 in differentiated primary spinal motor neurons after nutrient-starvation. Since this cell type is very sensitive to lysosomal inhibitors (data not shown), we avoided the use of such inhibitors in this experiment. As in fibroblasts, diffused as well as punctated immunostainings for p62 and LC3 in soma were both decreased after a transient starvation in the wild-type neuronal cells (Figure 9A). By contrast, the same treatment induced the enlargement of LC3/p62-double positive puncta/vesicles in soma of *Als2* ^−/−^ cells (Figure 9A). Quantitative analysis revealed that the basal level of p62 in *Als2* ^−/−^ cells was higher than that of wild-type cells (Figure 9B and 9C). Further, the levels of p62 in *Als2* ^−/−^ cells was unaltered by starvation, while those in wild-type were significantly decreased (Figure 9C and S12). Collectively, loss of ALS2 in cultured spinal motor neurons compromises autophagosomal-endolysosomal trafficking.

### SOD1 is partially colocalized with ALS2 and LC3 onto autophagosome-endosomal compartments in NSC-34 cells

Finally, to investigate the ALS2's contribution to the degradation of mutant SOD1, we analyzed the relative changes in the ectopically-expressed SOD1^H46R^ levels in cycloheximide-treated wild-type and *Als2* ^−/−^ fibroblasts by the treatment with either the UPS inhibitor (epoxomicin) or CQ according to the methods as described [37]. No significant differences in the SOD1^H46R^ levels between wild-type and *Als2* ^−/−^ cells were detected under the experimental conditions used (data not shown), indicating that effects of ALS2 on either the UPS- or lysosome-dependent SOD1^H46R^ degradation should be marginal, or undetectable in short-term cell-culture experiments.

**Figure 10:**
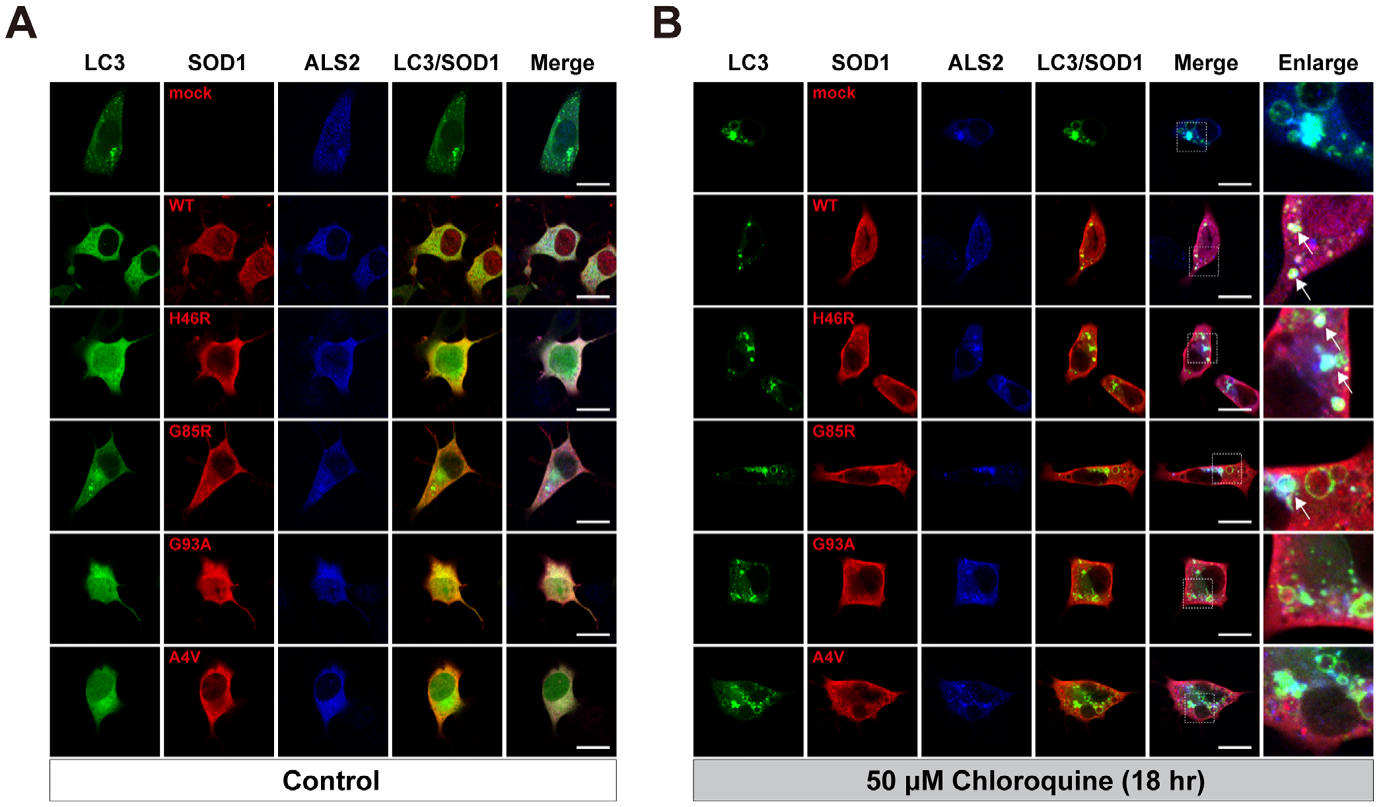
SOD1 is partially colocalized with ALS2 and LC3 onto autophagosomal/endolysosomal compartments in NSC-34 cells. (**A**) Ectopically expressed EGFP-LC3 (LC3), FLAG-tagged SOD1 (SOD1: SOD1^WT^; WT, SOD1^H46R^; H46R, SOD1^G85R^; G85R, SOD1^G93A^; G93A, SOD1^A4V^; A4V), and ALS2 were diffusedly distributed throughout the cytosol with no colocalization in NSC-34 cells under normal conditions (Control). (**B**) Under the treatment with 50 µM chloroquine for 18 hr, by which endolysosomal protein degradation was severely inhibited, ectopically expressed SOD1^WT^, SOD1^H46R^, and SOD1^G85R^ were partially colocalized with LC3/ALS2 onto enlarged endolysosomal vesicular compartments in NSC-34 cells (white arrows in enlarged images). Fourth and fifth columns display the merged images for double (LC3 and SOD1) and triple stainings, respectively (**A** and **B**). Sixth columns in **B** (Enlarge) represent a higher magnification of the merged-images of the respective 5^th^ columns in **B**. Scale bars = 10 µm.

To investigate the relationship between ALS2/LC3-localizing autophagosome-endosomal compartments and neuronal SOD1 dynamics in more detail, we conducted a series of co-transfection experiments in the presence of either the UPS or lysosomal inhibitor using NSC-34 motor neuron-like hybrid cell lines. Ectopically expressed EGFP-LC3, FLAG-tagged SOD1, and ALS2 were diffusedly distributed throughout the cytosol with no colocalization in NSC-34 cells under normal cultured conditions (Figure 10A). The treatment with the UPS inhibitor (2 µΜ MG132, 3hr) induced a formation of small punctated cytoplasmic aggregates of SOD1 mutants, while the distribution of EGFP-LC3 and ALS2 were unchanged (data not shown). On the other hand, the CQ treatment resulted in an extensive enlargement of ALS2/LC3-positve vesicular compartments in NSC-34 cells (Figure 10B). Intriguingly, ectopically expressed SOD1, namely SOD1^WT^ and SOD1^H46R^, were frequently colocalized with and accumulated onto such enlarged vesicular compartments (Figure 10B, white arrows in enlarged images), supporting the notion that a portion of cytoplasmic SOD1 is indeed degraded through the autophagy-endolysosomal system [37], [38].

## Discussion

**Figure 11:**
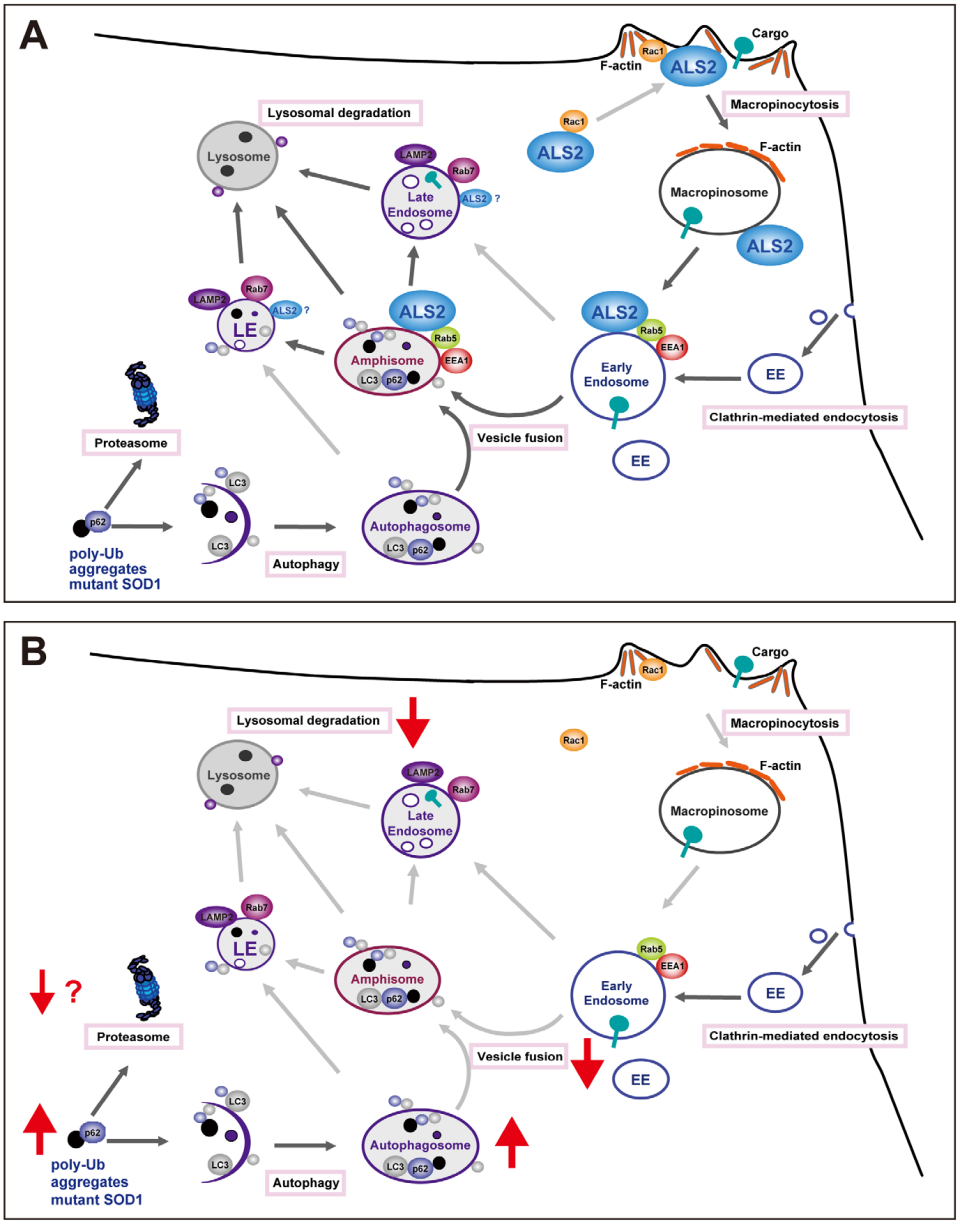
Proposed cellular functions of ALS2/alsin. (**A**) As previously reported [16], cytoplasmic ALS2 is recruited to membrane ruffles and then to macropinosomes via macropinocytosis upon Rac1 signaling. Subsequently, ALS2 localizing to nascent macropinosomes activates Rab5 and enhances the recruitment of EEA1, PI3K/Vps34, and souble *N*-ethylmaleimide-sensitive factor attachment protein receptor (SNARE) protein complex (not shown), thereby promoting the maturation (fusion and trafficking) of macropinosomes and early endosomes (EE). In the present study, we show that ALS2 is also present to the p62/LC3-positive autophagosomes in cells, suggesting that ALS2 plays a role not only in the maturation of macropinosomes and EE, but also of autophagosomes via their heterotypic fusions, generating amphisomes. The resulting amiphisomes further mature to late endosomes (LE) accompanying the recruitment of Rab7, and ultimately fuse with lysosomes, thereby cargo molecules including poly-ubiquitinated proteins are degraded. Poly-ubiquitinated proteins associated with p62 are also degraded by the proteasomes. (**B**) Loss of ALS2 results in a decrease in endosome fusion, thereby the maturation of autophagosomes and endolysosomal trafficking are disturbed.

ALS2/alsin is an activator for the small GTPase Rab5 [15], and involves not only in early endosome/macropinosome trafficking and fusion [16], [17] but also in neuroprotection against MND-associated pathological insults, such as toxicity induced by mutant SOD1 [25], [46]. However, molecular mechanisms underlying the relationship between ALS2-associated cellular function and its neuroprotective role remain unclear. In this study, we demonstrated that disturbance of endolysosomal trafficking by ALS2 loss exacerbated the SOD1^H46R^-mediated neurotoxicity by accelerating the accumulation of immature vesicles and insoluble proteins in the spinal cord. Thus, ALS2 might play a role in endolysosomal trafficking *in vivo*, accounting for the ALS2-assocaited neuroprotective function (Figure 11).

It has been reported that overexpression of ALS2 protects cultured motor neuronal cells from toxicity induced by SOD1 mutants; A4T, G85R, and G93R [25], [46]. This protection is dependent on the physical interaction between mutant SOD1 and ALS2 [25], and such ALS2-mediated neuroprotective function exerts through the activation of the Rac1/phosphatidylinositol 3-kinase (PI3K)/Akt prosurvival pathway [46]. However, we were unable to reconfirm the ALS2 interaction with either wild-type or SOD1 mutants; H46R, A4V, G93A, and G85R (data not shown), and the Rac1 activation by ALS2 [16]. In addition, although we revealed a milder accumulation of wild-type as well as mutant SOD1 onto ALS2/LC3-double positive autophagosome-endosomal compartments in motor neuron-like cells under dysfunctional lysosomal conditions, ALS2 *per se* seems to play a limited role in the SOD1 degradation in cultured cells (data not shown). Nonetheless, a significant accumulation of insoluble mutant SOD1, polyubiquitinated proteins, p62, and LC3-II emerged in the spinal cord of ALS2 deficient *SOD1^H46R^* mice at an early- or even pre-symptomatic stage. A large number of studies have suggested that overexpression of mutant SOD1 results in the increased levels of misfolded and aggregated proteins [41], which compromises the UPS for protein degradation [39], and initiates the ER-associated unfolded protein response (UPR) [47]. Under such stress conditions, autophagy-lysosomal protein degradation appears to function as a safeguard for maintaining the cellular homeostasis [48], [49]. Thus, it is possible that the persistent and long-term excess production of SOD1^H46R^, which exceeds the intracellular capacity for the clearance of misfolded proteins by the UPS and/or the autophagy-endolysosomal system, results in the accumulation of insoluble proteins in tissues, and that loss of ALS2 accelerates such phenotypes *in vivo*.

Intriguingly, we here showed that the proteasome activity was gradually induced rather than impaired in the spinal cord of SOD1^H46R^-expressing mice as the disease progressed, and that loss of ALS2 further enhanced their activation. A recent study has demonstrated that Ub^G76V^-GFP reporter-expressing *SOD1^G93A^* mice show only mild proteasome impairment in motor neurons at symptomatic stage but not before symptoms onset [39]. These results suggest that the UPS impairment is not a primary cause for the accumulation of the insoluble proteins, while the possibility that the UPS is impaired by perturbing the delivery of ubiquitinated substrates to the proteasome without affecting the proteasome activity [50] should not be excluded. On the other hand, both p62 and LC3-II are localized onto autophagosomes and are preferentially degraded by the endolysosomal system [36], [50], [51]. Thus, either the excess autophagosome formation or the overwhelmed endolysosomal system, or both of them, can account for the observed accumulation of the insoluble proteins such as p62 and LC3-II in SOD1^H46R^-expressing mice. Since ALS2 appears to regulate endolysosomal protein degradation rather than autophagosome formation, we favor the hypothesis that loss of ALS2 augments the SOD1^H46R^-mediated neurotoxicity, albeit its exact entity is still unclear, by slowing the fusion-mediated endolysosomal trafficking and/or autophagic clearance of misfolded protein aggregates (Figure 11). Contrary to our findings, a recent study has reported the enhanced endolysosomal degradation of glutamate receptors in *Als2* ^−/−^ cells [44]. The reason for this discrepancy is currently unclear. It may be due to that the fates (degradation and/or recycling) of membrane bound functional molecules such as receptors and misfolded/ubiquitinated non-functional protein aggregates are differently regulated within cells. Indeed, this notion is supported by the recent findings that loss of histone deacetylase 6 (HDAC6) shows seemingly opposite effects on endolysosome-dependent protein degradation in cells; HDAC6 loss results in a failure of autophagosome maturation on the one hand [52], but also promotes EGF receptor degradation on the other [53].

Given the role of ALS2 on early endosomal and/or macropinosomal compartments as an activator for Rab5 [15], [16], how does ALS2 regulate rather downstream endolysosomal pathway? It has been demonstrated that autophagosome maturation is essential for the degradation of abnormal proteins and organelle via the autophagy-lysosome pathway [54]. This maturation is accomplished by the sequentially fusion of nascent autophagosomes with late endosomes and lysosomes under the control of many regulatory factors including endosomal sorting complex required for transport (ESCRT) and small GTPase Rab7 [54]. Remarkably, it has recently been revealed that the fusion of autophagosomes with functional early endosomes and endosomal coatomer is also required for autophagy-dependent protein degradation [55]. Since ALS2 is colocalized with LC3 and p62 on autophagosome/endosome hybrid membrane compartments called amphisomes [43], it is conceivable that ALS2 contributes to the much earlier phase of the autophagosome maturation, i.e. amphisome formation, via regulating the fusion between early endosomes and nascent autophagosomes (Figure 11).

An EM analysis revealed an extensive degeneration of spinal axon with accompanying the accumulation of granular/osmiophilic aggregates and autophagosome-like vesicles in early symptomatic *SOD1^H46R^* mice, while spinal motor neurons were still preserved. This indicates that motor dysfunction seen in these mice is primarily associated with axonal degeneration rather than motor neuron loss. Importantly, loss of ALS2 appears to aggravate such pathological phenotypes in this mice model. In cultured neurons from wild-type mice, ALS2 is enriched to the membrane and vesicular compartments at the distal tip [17] as well as at the branching points (Otomo et al., unpublished) of neurites, and acts as sustenance in axonal development and function [17]. Interestingly, it has been reported that Rab5 regulates an early sorting step preceding axonal transport, while Rab7, a regulator for the endosome/autophagosome-lysosome fusion, regulates long-rage retrograde axonal transport in motor neurons [56]. Together with our findings, ALS2 may regulate the early step of endosome and/or autophagosome maturation through the activation of Rab5 within the axons, and loss of ALS2 results in an increased number of the immature vesicles, which precludes the normal long-range axonal vesicle trafficking by Rab7, leading to the accumulation of MVBs and autophagosome-like vesicles, axonal swelling, and degeneration in *SOD1^H46R^* mice.

In this study, we demonstrated that loss of ALS2 resulted in a significant increase in the levels of astrocytic intermediate filaments, namely those of vimentin from a pre-symptomatic stage. It has been reported that vimentin is also expressed in motor neurons, and its expression is upregulated in a number of animal models for MNDs as the disease progresses [57]. Thus, it is still possible that neuronal vimentin is responsible for such increments. However, our preliminary immunohistochemical analysis of *SOD1^H46R^* and *Als2* ^−/−^;*SOD1^H46R^* mice, in which the increased levels of vimentin were mainly observed in astrocytes rather than motor neurons in the spinal cord (Otomo et al., unpublished), did not fit this notion. As there is currently no supportive evidence that ALS2 is expressed in glial cells including astrocyte [15], [19], loss of ALS2 may lead to insidious adverse effects on neuronal cells, which in turn initiates and accelerates astrogliosis through some signaling cross-talk between neurons and astrocytes. Interestingly, a recent study has shown that ALS2 depleted spinal motor neurons, but not cortical neurons, are rescued by co-cultured astrocytes, indicative of a cell-type specific neuron-glia crosstalk [19]. Further investigation of such neuron-astrocyte interaction on the pathogenesis is warranted.

One important question arising from this study is whether the effects of ALS2 loss are specific to H46R mutation in SOD1 or not. Previously, it has been reported that loss of ALS2 does not affect the pathological course of *SOD1^G93A^* mice [29], [30]. Although the exact reasons for this discrepancy are unclear, pathological differences observed between *SOD1^H46R^* and *SOD1^G93A^* mice might be related. Despite that the expression levels of mutant SOD1; ∼20-fold of endogenous SOD1, were comparative between *SOD1^H46R^* and *SOD1^G93A^* mice (data not shown), the pathology of the spinal cord was very different. In *SOD1^G93A^* mice, a prominent distension of mitochondria forming vacuolar structures with accompanying much faster progression of disease symptoms was evident [58], while such pathological features were barely observed in *SOD1^H46R^* mice. Rather, a widespread axonal degeneration with preserved motor neuron was noticeable in the spinal cord of *SOD1^H46R^* mice. This suggests that molecular basis for the pathogenesis in each mutant SOD1-expressing model may not be the same. Recent findings that dynein mutants crossed with three different SOD1 mutant animals showed different outcomes [59] also support this notion. We are currently speculating that overlapping pathogenic processes between *SOD1^H46R^* and *Als2* null mice; i.e., the axonal degeneration in the spinal cord [6], [21], [31], are implicated in the enhancement of the additive adverse effects. By contrast, a devastating mitochondrial dysfunction in *SOD1^G93A^* mice overwhelms the modest symptoms by the ALS2 deficiency.

In conclusions, loss of ALS2 impairs the maturation of autophagosomes, which causes the lowering of the autophagic flux, thereby accumulating immature vesicles and insoluble misfolded proteins under stress conditions. Thus, disturbance of the autophagosome-endolysosomal trafficking by ALS2 loss might be a causative of the manifestations of *ALS2*-linked MNDs in human. Notably, functional impairment of other endolysosomal-associated proteins such as ESCRT subunit and Rab7 also causes motor neuron dysfunction [60], [61]. Further characterizations will give us more clues to understanding physiological roles of autophagy-endolysosomal process in the pathogenesis for ALS and other MNDs.

## Materials and Methods

### Plasmids

We generated pEGFP-LC3B by subcloning the RT-PCR-amplified cDNA from mouse brain mRNA. The DNA sequence of the insert as well as its flanking regions in the plasmid construct was verified by sequencing. Previously generated ALS2 construct; pCIneo-hALS2_L [15] was also utilized. Four SOD1 constructs; pcDNA3-FLAG-SOD1-WT, pcDNA3-FLAG-SOD1-G93A, pcDNA3-FLAG-SOD1-A4V, pcDNA3-FLAG-SOD1-G85R, were kindly provided by Dr. Ryosuke Takahashi (Kyoto University). We additionally generated pcDNA-FLAG-SOD1-H46R by subcloning the RT-PCR-amplified cDNA fragment from *SOD1^H46R^* mice brain mRNA.

### Antibodies

Primary antibodies used for western blot analysis included two independent rabbit polyclonal anti-ALS2; HPF1-680 (1∶3,000) [15] and MPF1012-1651 (1∶3,000) [62], rabbit polyclonal anti-SOD1 (FL-154) (1∶20,000, Santa Cruz), mouse monoclonal anti-ubiquitin (P4D1) (1∶3,000, Santa Cruz), guinea pig polyclonal anti-p62/SQSTM1 (1∶6,000, Progen), rabbit polyclonal anti-LC3 (1∶5,000, MBL), rabbit polyclonal anti-peripherin (1∶5,000, CHEMICON), mouse monoclonal anti-NFH (1∶12,000, Sigma), rabbit polyclonal anti-TDP-43 (1∶2,000, Protein Tech Group), mouse monoclonal anti-Hsp70 (1∶3,000, Santa Cruz), rabbit polyclonal anti-proteasome 20S subunit alpha-5 (1∶3,000, Thermo Scientific), rabbit polyclonal anti-proteasome 20S C2 (1∶3,000, Thermo Scientific), rabbit polyclonal anti-proteasome 20S LMP2 (Novus), mouse monoclonal anti-vimentin (1∶3,000, Sigma), rabbit polyclonal anti-GFAP (1∶50,000, Biomeda), mouse monoclonal anti-β-tubulin (1∶100,000, CHEMICON), mouse monoclonal anti-α-tubulin (1∶10,000, Sigma), and mouse monoclonal anti-glyceraldehyde 3-phosphate dehydrogenase (GAPDH) (1∶10,000, CHEMICON) antibodies. Secondary antibodies included horseradish peroxidase (HRP)-conjugated goat anti-guinea pig IgG (1∶5,000, Santa Cruz), donkey anti-rabbit IgG (1∶5,000, Amersham Bioscience), and sheep anti-mouse IgG (1∶5,000, Amersham Bioscience) antibodies.

Antibodies used for immunohistochemical and immunocytochemical studies included rabbit polyclonal anti-ALS2; HPF1-680 (1∶5,000), rabbit polyclonal anti-SOD1 (1∶500, MBL), guinea pig polyclonal anti-p62/SQSTM1 (1∶1,000, Progen), rabbit polyclonal anti-ubiquitin (1∶200, DakoCytomation), rabbit polyclonal anti-LC3 (1∶1,000, MBL), rabbit polyclonal anti-MAP2 (1∶1,000, CHEMICON), mouse monoclonal anti-GFAP (1∶500, CHEMICON), anti-myelin basic protein (MBP) (1∶5,000, GeneTex), mouse monoclonal anti-EEA1 (1∶100, BD biosciences), mouse monoclonal anti-LAMP2 (1∶250, BD biosciences), and mouse monoclonal anti-FLAG-M2 (1∶500, Stratagene) antibodies. Secondary antibodies included Alexa 594- and Alexa 647-conjugated goat anti-guinea pig IgG (1∶500, Invitrogen), Alexa 488-, Alexa 594-, and Alexa 647-conjugated anti-rabbit IgG (1∶500, Invitrogen), and Alexa 488-, Alexa 594-, and Alexa 647-conjugated anti-mouse IgG (1∶500, Invitrogen) antibodies.

### Animals

We generated *SOD1^H46R^* transgenic mice on an *Als2*-null background by crossing *Als2* ^−/−^ mice [20] with the *SOD1^H46R^* mouse line expressing familial ALS-linked SOD1^H46R^ under the control of inherent human *SOD1* promoter [31]. We first generated congenic lines of both *SOD1^H46R^* transgenic and *Als2* ^+/−^ mice by each backcrossing more than 10 generations with C57BL/6N (B6) mice. Next, we produced *Als2* ^+/−^;*SOD1^H46R^* mice by crossing male *SOD1^H46R^* and female *Als2* ^+/−^ mice, and then generated mice with six different genotypes; *Als2* ^+/+^ (wild-type), *Als2* ^+/−^, *Als2* ^−/−^, *Als2* ^+/+^;*SOD1^H46R^*, *Als2* ^+/−^;*SOD1^H46R^*, and *Als2* ^−/−^;*SOD1^H46R^*, by crossing male *Als2* ^+/−^;*SOD1^H46R^* and female *Als2* ^+/−^ mice. *SOD1^G93A^* mice on a B6 background were generated by crossing B6SJL-TgN(SOD1-G93A)1Gur males (Jackson Laboratory) to B6 females for 4–7 generations (N4–N7), and then *SOD1^G93A^* mice on an *Als2*-null background were generated in a similar manner. We further generated transgenic mice expressing human full-length *ALS2* transcript under the control of the human *ALS2* promoter by microinjecting the construct into fertilized eggs from B6 mice. The construct was generated by connecting six DNA fragments originated from human *ALS2*; two genomic fragments, spanning −2463_IVS+2307 (4.5 kb; promoter + exon 1 + part of intron 1) and IVS1-773_IVS1-1 (0.8 kb; part of intron 1), 5′ untranslated region (UTR) of exon 2 (123 bp), the full-length *ALS2* cDNA (5 kb; open-reading frame; ORF), and the 3′ UTR (1.3 kb), and a 30 bp of downstream element (DE) (Figure S3A). All lines of *ALS2* transgenic (*ALS2*-tg) mice were viable and fertile with no evidence for abnormalities including their lifespan (∼2.5 yr). The offsprings were genotyped by PCR using genomic DNA from tail tissue. Mice were housed at an ambient temperature of 22°C with a 12 hr light/dark cycle. Food and water were fed *ad libitum*. Body weight of each animal was weekly monitored. Their lifespan (endpoint) was determined by the observations that mice were unable to move by themselves. All animal experimental procedures were approved by The Institutional Animal Care and Use Committee at Tokai University.

### Behavioral analysis

Motor coordination and balance was assessed by a balance-beam test using the fixed-stainless steel bar (45 cm long and 0.9 cm in diameter) at 12 weeks of age, and weekly thereafter until the day at which mice were unable to stay on the bar. Each mouse was given five trials, and the maximum durations (up to 60 sec) at which mice fall off from the bar were scored. To evaluate the spontaneous motor activities in mice, we conducted rearing and cage activity tests by using SUPERMEX with an infrared ray sensor monitor (Muromachi Kikai). Both rearing and cage activities were uninterruptedly monitored for 7 consecutive days starting at either 12 weeks or 18 weeks of age. The cumulative counts of rearing and cage activities for either a light- (12 hr; 7:00–19:00) or a dark-period (12 hr; 19:00–7:00) were analyzed.

### Southern blot analysis

The probe DNA spanning the region between introns 3 and 5 for human *SOD1* gene (product size; 1,955 bp) was prepared by PCR amplification using primer sets as follows; hSOD1_probe_L; 5′- CCCCTGCTCCCAAATGCTGGAATGC-3′, hSOD1_probe_R; 5′- GGGGCCTCAGACTACATCCAAGGG-3′. Genomic DNA samples prepared from tail tissues were digested with *Fba*I, separated by electrophoresis, and blotted onto nylon membrane (Hybond-N+; Amersham Biosciences). The blot was hybridized with [α^32^P-dCTP]-labeled hSOD1_probe, detecting an ∼3.3 kb restriction fragment of the transgene; *SOD1^H46R^*. As a control, mouse *Actb* cDNA encoding β-actin was used as a probe.

### Tissue sample preparation for western blot analysis

Brain and spinal cord tissues were homogenized in Lysis buffer A [25 mM Tris-HCl (pH 7.5), 50 mM NaCl, 1% (w/v) Triton X-100 (TX), Complete Protease Inhibitor Cocktail (Roche)], and were centrifuged at 23,000*g* for 20 min at 4°C. The resultant supernatant was collected as a TX-soluble fraction. The insoluble pellet fraction was once washed with A buffer, and then suspended with Lysis buffer B [25 mM Tris-HCl; pH 7.5, 50 mM NaCl, 5% (w/v) sodium dodecyl sulfate (SDS)], sonicated, and left for 30 min at room temperature (RT). After the centrifugation at 23,000*g* for 20 min, the supernatant was collected as a TX-insoulble/SDS-soluble fraction. Fibroblasts and HeLa cells were harvested and lysed in Lysis buffer C [50 mM Tris-HCl (pH 7.5), 150 mM NaCl, 0.1% SDS, 1% TX, 0.5% sodium deoxycholate, and Complete Protease Inhibitor Cocktail (Roche)], and sonicated. Protein concentration of each fraction was determined by the Micro BCA system (Pierce).

### Western blot analysis

Equal amount of protein (1–2 µg) from each fraction was subjected to SDS-polyacrylamide gel electrophoresis, and transferred onto a polyvinylidene difluoride membrane (Bio-Rad). The membranes were blocked with Blocking One (Nacalai Tesque) for 1 hr at RT, incubated with the primary antibody in TBST [20 mM Tris-HCl (pH 7.5), 150 mM NaCl, 0.1% Tween 20] containing 5% Blocking One (Nacalai Tesque). After washing with TBST, membranes were incubated with HRP-conjugated secondary antibody. Signals were visualized by Immobilon™ Western (Millipore) and BioMax X-ray films (Kodak). The bands or signal intensities were quantified by analyzing the digitally-captured images using CS Analyzer ver3 (ATTO).

### Quantitative reverse transcriptase-PCR (qRT-PCR)

Total RNA was extracted from lumbo-sacral cord using Sepasol-RNAI (Nakarai Tesque), and purified by SV Total RNA Isolation System (Promega) according to manufacturer's instructions. The qRT-PCR was performed on a 0.5 µg of total RNA using QuantiFast™ SYBR Green RT-PCR (Qiagen) with specific primers (0.6 µM each) as follows; human *SOD1*; forward (F): 5′-AGGGCATCATCAATTTCGAG-3′, reverse (R): 5′-ACATTGCCCAAGTCTCCAAC-3′), *Sqstm1* (F: 5′-CGGTGAAGGCCTATCTTCTG-3′, R: 5′-TGTCAGCTCCTCATCACTGG-3′), *Map1lc3a* (F: 5′-TGCCTGTCCTGGATAAGACC-3′, R: 5′-CCGTCTTCATCCTTCTCCTG-3′), *Map1lc3b* (F: 5′-CCGAGAAGACCTTCAAGCAG-3′, R: 5′-ACACTTCGGAGATGGGAGTG-3′), *Gfap* (F: 5′-GCTTCCTGGAACAGCAAAAC-3′, R: 5′-GCAAAGTTGTCCCTCTCCAC-3′), *Vim* (F: 5′-ACCAGGTCTGTGTCCTCGTC-3′, R: 5′-AATAGAGGCTGCGGCTAGTG-3′), and *Gapdh* (F: 5′-AACTTTGGCATTGTGGAAGG-3′, R: 5′-CACATTGGGGGTAGGAACAC-3′). The levels of all transcripts were normalized for the *Gapdh* mRNA level in each sample.

### 20S proteasome activity assay

Spinal cord tissues were homogenized in Lysis buffer D [50 mM HEPES (pH 7.5), 5 mM EDTA, 150 mM NaCl, 1% (w/v) TX], and were centrifuged at 23,000*g* for 20 min at 4°C. Protein concentration of the resultant supernatant was determined by the Micro BCA system (Pierce), and adjusted to 2 µg protein/µl with Lysis buffer D. A 10 µl of each sample (20 µg) was subjected to the proteasome activity assay using 20S Proteasome Activity Assay Kit (Chemicon) according to manufacturer's instructions. The lactacyctin (25 µM)-inhibitable chymotrypsin-like proteasome activity was measured by quantitating the fluorescent intensity for the fluorophore 7-amino-4-methylcoumarin (AMC) hydrolyzed from the substrate peptide; LLVY-AMC using a 360/460 nm filter set in a fluorometer (CytoFluor 4000; PerSeptive Biosystems).

### Histological analysis

Mice were anesthetized with 4% halothane in a mixture of N_2_O/O_2_ (70∶30), and transcardially perfused with physiological saline containing 1,000U/ml heparin, followed by 2% paraformaldehyde (PFA)/2% glutaraldehyde (GA) in 0.1 M phosphate buffer (PB) (pH 7.3). Brain and spinal cord was removed and post-fixed with the same fixative for 12 hr or 36 hr at 4°C and with 2% GA for 2 hr at 4°C, followed by washing with 0.1 M PB (pH 7.3). Lumbar segment was dissected out and post-fixed in 1% osmium tetroxide in 0.05 M PB (pH 7.4). After dehydration in graded alcohol, the tissues were embedded in the epoxy resin. Semi-thin sections (2–3 µm) of L5 lumbar cord were stained with 0.5% toluidine blue and examined under a computer-assisted light microscope (BZ-9000, KEYENCE). Selected areas of the spinal cord were cut into ultrathin sections, and stained with uranyl acetate and lead citrate for ultrastructural examination using electron microscopes (H-7100, Hitachi; JEOL1200EX, JEOL).

### Immunohistochemical analysis

Anesthetized mice were transcardially perfused with 4% PFA in 0.1 M PB (pH 7.5). Brain and spinal cord were removed and post-fixed for at least 48 hr in 4% PFA followed by paraffin embedding. For fluorescent immunohisochemistry, 6 µm paraffin embedded sections were cut on a microtome, and brain and spinal cord sections were incubated in phosphate buffered saline (PBS, pH 7.4) with 5% normal goat serum (NGS) and 0.1% TX for 1 hr at RT. For double- or triple-immunostaining, sections were incubated with primary antibodies in PBS containing 0.05% TX overnight at 4°C. Sections were incubated with secondary antibody for 3 hr at RT. Controls for all immunostainings were performed simultaneously by omitting the primary antibody. Sections were coverslipped using VectorShield (Vector Laboratories) with or without 4′,6-diamidino-2-phenylindole dihydrochloride (DAPI) for nuclei counterstaining, and analyzed by a computer-assisted light microscope (BZ-9000, KEYENCE), or Leica TCS-NT system (Leica Microsystems) and processed by ImageJ 1.39u (NIH). All images presented are representative of at least n = 2–3 animals examined in each group at each time point.

### Cell culture

HeLa (CCL-2, ATCC) and NSC-34 (CELLutions Biosystems) cells were cultured in Dulbecco's modified Eagle's medium (DMEM) supplemented with 10% heat inactivated fetal bovine serum (FBS) (Invitrogen), 100 U/ml penicillin G, and 100 µg/ml streptomycin.

Primary fibroblast cultures were established from E14–E15 mouse embryos. The skin tissues were isolated from pups, washed with ice-cold Hank's balanced salt solution devoid of calcium and magnesium ion [HBSS(-)] (Invitrogen) and treated with 0.5 ml of 0.25% trypsin-EDTA for 15 min at 37°C. Trypsin-EDTA was removed and washed several times with HBSS(-). Tissue samples were then treated with DNase I (final 50 µg/ml) in DMEM supplemented with 10% FBS, 100 U/ml penicillin, and 100 µg/ml streptomycin at RT for 10 min. The dissociated cells were seeded onto a T75 flask at an appropriate cell density, and cultured in the same culture medium.

Primary hippocampal neuronal cultures were established from E18 embryos. In brief, tissues from each embryo were dissected out and immediately placed into 1 ml of ice-cold HBSS(-). After removing HBSS(-) by aspiration, 0.5 ml of 0.25% trypsin-EDTA was added and incubated for 15 min at 37°C. Trypsin-EDTA was removed and washed several times with 20% FBS/Neurobasal medium (Invitrogen). Tissue samples were treated with DNase I (final 50 µg/ml) in 20% FBS/Neurobasal medium for 10 min at RT. After the centrifugation at 150*g* for 15 s, the resulting tissue pellets were dissociated in a 0.6 ml of Neurobasal medium containing 20% FBS by pipeting using the fire polished Pasteur pipet. After counting the living cell numbers by the trypan blue assay, the cells were plated onto poly-D-lysine coated round glasses at a density of 10^5^ cells/mm^2^ (days *in vivo* 1; DIV1) for transfection in neuronal cell culture (NCC) media [Neurobasal medium containing 1×B27 supplement (Invitrogen), 25 µg/ml insulin (Sigma), 0.5 mM L-glutamine, 50 µg/ml streptomycin, and 50 U/ml penicillin G], and cultured for 12 hr at 37°C. Medium was exchanged with the fresh one, and cultured for another 36 hr. Medium was further replaced with the fresh NCC medium containing cytosine β-D-arabinofuranoside hydrochloride (Ara-C; Sigma).

Primary spinal neuronal cultures were established from the lumbar spinal cord of E14 embryos. In brief, after removal of the dorsal root ganglia and meninges under microscopic observation, the ventral horns were dissected using microscalpels, transferred to a 1.5-ml tube containing 0.5 ml of HBSS(-)/0.025% (w/v) of trypsin, and incubated for 20 min at 37°C. After the trypsin treatment, 0.5 ml of HBSS(-) was added to the tube. The tissues were dissociated by gentle pipetting, and then centrifuged at 400*g* for 10 min at RT. The pellets were suspended in 0.5 ml of HBSS(-). The resulting suspension was centrifuged through a bovine serum albumin (BSA) cushion [3% (w/v) in HBSS(-)] at 700*g* for 10 min at 4°C. The cells obtained at this step represent mixed neuron/glia population. The cells were resuspended in Neurobasal medium containing physiological concentrations of Ca^2+^ (1.8 mM) and Mg^2+^ (0.8 mM) (Invitrogen) and centrifuged at 1,000*g* for 10 min at RT. The pellet was suspended in Neurobasal medium (∼400 µl) containing 1×B27, 0.5 mM L-glutamine, 2% FBS, 25 µM 2-mercaptoethanol, 25 µM glutamate, 50 U/ml penicillin, 50 µg/ml streptomycin, and 10 ng/ml Brain-derived neurotrophic factor (BDNF), and seeded onto poly-D-lysine (Sigma) pre-coated glass cover slips in the wells of a 24-well plate at a density of 5,000 cells/mm^2^. After 24 hr, the medium was replaced with 400 µl of fresh Neurobasal medium without glutamate, thereafter 100 µl of the same medium was added three times a week to the cultures for 2 weeks until the fixation.

### Transfection

Transfection was performed by using Effectene Transfection Reagent (Qiagen), Lipofectoamine 2000 (Invitrogen), or Lipofectamine™ LTX Reagent (Invitrogen) according to the manufacturer's instructions. HeLa and primary neuronal cultures were transfected with plasmid DNAs as previously described [15], [20]. NSC-34 cells were seeded onto a 24 well-plate at a density of 2×10^5^ cells, cultured for 16 hr, and transfected with an appropriate amount of plasmid DNAs. After 24 hr of culture, cells were trypsinized and re-seeded onto a 11 mm round glass cover slip coated with 0.01% poly-D-lysine and 10 µg/µl laminine at a density of 5×10^4^ cells/well. Finally, cells were cultured in DMEM with 10% FBS for 18 hr in the presence or absence of CQ (50 µM) or MG132 (10 µM), or a solvent alone (dimethyl sulfoxide; DMSO) as a control.

### Nutrient starvation

Fibroblasts and HeLa cells were seeded onto a 6 well-plate at a density of 2×10^5^ cells, and cultured for 24 hr. In case of primary spinal neurons, dissociated tissues were seeded onto a poly-D-lysine-coated cover slip and incubated for 14 days (DIV14) under appropriate conditions (see above). Medium was then exchanged with Earle's Balanced Salt Solution (EBSS) (Sigma) lacking FBS, and cells were cultured for another 6–8 hr either in the presence or absence of pepstatin A (20 µg/ml) or CQ (0.5 µM or 12.5 µM).

### Small Interfering RNA-mediated knockdown of ALS2

To knock down the expression of the endogenous *ALS2* gene in HeLa cells, we conducted the oligonucleotide-based RNA interference using small interfering RNA (siRNA). In brief, 0.4 µl siRNA (2.5 µM) [ALS2si-1 (SI00128226; QIAGEN), ALS2si-2 (SI00128219; QIAGEN), ALS2si-3 (SI00128205; QIAGEN), ALS2si-4 (SI00128212; QIAGEN), and control (scrambled siRNA, cat#4611; Ambion)] and 0.8 µl RNAiMAX (Invitrogen) were mixed with 100 µl OPTI-MEM, and incubated for 20 min at RT. One-hundred µl of the resulting mixture was transferred onto a 24 well-plate, and mixed with 400 µl of medium containing 2.5×10^4^ dissociated HeLa cells with a final siRNA concentration of 1 nM. After 48 hr of culture, cells were harvested, lysed in Lysis buffer C, and sonicated. Equal amount of protein from each sample was subjected to western blot analysis.

### Immunocytochemistry

Cells were washed with PBS(-) twice, fixed with 4% PFA in PBS(-) (pH 7.5) for 20 min, followed by permeabilization with 0.3% TX in PBS(-) for 20 min or with 100 µg/ml digitonin for 30 min at RT. The primary antibody, diluted in PBS(-) containing 1.5% normal goat serum and 50 µg/ml digitonin, was added to cells and incubated overnight at RT. Appropriate secondary antibodies were used for the detection of the signals of either the tag epitope or proteins of interest. Finally, images of optical sections with 0.025 µm thickness were captured and analyzed by Leica TCS_NT confocal-microscope systems (Leica Microsystems). Randomly selected images for spinal neurons were digitally-processed by ImageJ 1.39u (NIH) to demarcate the outline of the cell body, and fluorescent intensities for p62 signals within the demarcated area (corresponding to the cell body for the single cell) were measured using the fluorescence microscopy (Leica) with identical settings.

### Statistical analysis

Statistical analyses were conducted using Statview 5 (SAS Institute) or PRISM 5 (GraphPad). Statistical significance was evaluated by ANOVA followed by appropriate *post hoc* tests for multiple comparisons between groups. Survival data were compared using Kaplan-Meier survival analysis with Log-rank (Mantel-Cox) test. A *p*-value <0.05 was considered as reaching statistical significance.

## Supporting Information

Figure S1 **Copy numbers of the transgene in human** ***SOD1^H46R^*** **transgenic mice on different** ***Als2*** **genotypes used were comparable.** (**Upper panel**) Image for the ethidium bromide-stained mouse genomic DNA digested with *Fba*I. Mice with five different genotypes [*Als2* ^+/+^;*SOD1^H46R^* (blue), *Als2* ^+/−^;*SOD1^H46R^* (green), *Als2* ^−/−^;*SOD1^H46R^* (red), *Als2* ^−/−^ (orange), and wild-type (WT) (black), (each n = 4)] were analyzed. Equal amount of genomic DNA (2 µg) was loaded and separated by agarose gel electrophoresis. The positions of size-markers are shown on the left. (**Middle panel**) Southern blot analysis of the mouse genomic DNA. The *Fba*I blot was probed with the radio-labeled human genomic DNA fragment of the *SOD1* gene. A 3.3 kbp of the restriction fragment originating from human *SOD1^H46R^* transgene was specifically detected. (**Lower panel**) As a control, the *Fba*I blot was re-probed with the radio-labeled mouse *Actb* (β-actin) cDNA, detecting two fragments originating from mouse endogenous *Actb* gene (2.0 and 1.4 kbp).
(1.18 MB PDF)

Figure S2 **Growth curves for** ***Als2*** **^−/−^ mice and effect of ALS2 loss on survival in** ***SOD1^G93A^*** **mice.** (**A**) Growth curves for female mice [wild-type (WT) (black circle; n = 16–36) and *Als2* ^−/−^ (orange square; n = 32–56)], and (**B**) for male mice [WT (n = 42–49) and *Als2* ^−/−^ (n = 46–55)]. (**A**–**B**) There were no differences in the mean values between WT and *Als2* ^−/−^ mice at any ages. Values are mean ± SD. Statistical significance is evaluated by ANOVA with Tukey's *post hoc* test. (**C**) Survival curves for *Als2* ^+/+^;*SOD1^G93A^* [blue circle; n = 14 (female; n = 2, male; n = 12)], *Als2* ^+/−^;*SOD1^G93A^* [green square; n = 25 (female; n = 13, male; n = 12)], and *Als2* ^−/−^;*SOD1^G93A^* [red triangle; n = 12 (female; n = 8, male; n = 4)]. Kaplan-Meier analysis identified significant difference between *Als2* ^+/−^;*SOD1^G93A^* and *Als2* ^−/−^;*SOD1^G93A^* (Log-rank test; *p* = 0.0479), while no significance between *Als2* ^+/+^;*SOD1^G93A^* and *Als2* ^−/−^;*SOD1^G93A^* was detected.
(0.36 MB PDF)

Figure S3 **Generation of human full-length** ***ALS2*** **expressing mice.** (**A**) Schematic representation of the *ALS2* transgenic construct. Human full-length *ALS2* transcript is expressed under the control of the human *ALS2* promoter. (**B**) Western blot analysis of ALS2 expression in cerebral cortex, cerebellum, and spinal cord from wild-type (WT) and 4 independent *ALS2*-tg lines; L6-2, L6-1, L31, and L34-1. Equal amount of protein from 1% Triton X-soluble fractions (5 Î¼g) was loaded in each lane, and anti-ALS2 polyclonal antibody (HPF1-680) was used to probe ALS2 (180 kDa) as indicated on the right. Upper and lower panels represent images for short and long exposures, respectively. The positions of size-markers are shown on the left.
(0.29 MB PDF)

Figure S4 **Quantitative analysis of the transcripts in the spinal cord.** The expression levels of (**A**) the human *SOD1^H46R^* transgene, (**B**) *Sqstm1* (p62), (**C**) *Map1lc3a* (LC3A), (**D**) *Map1lc3b* (LC3B), (**E**) *Gfap*, and (**F**) *Vim* (vimentin) genes, which are normalized by the level of *Gapdh*, in the lumbo-sacral cord from 18, 20, and 23 week-old mice with four distinct genotypes; wild-type (WT), *Als2* ^−/−^, *Als2* ^+/+^;*SOD1^H46R^*, and *Als2* ^−/−^;*SOD1^H46R^* are analyzed. Values are mean ± SEM (n = 3–5) in an arbitrary unit relative to 18 week-old wild-type mice except for the *SOD1^H46R^* expression in which values relative to 18 week-old *Als2* ^+/+^;*SOD1^H46R^* mice are shown. Statistical significance is evaluated by ANOVA with Bonferroni's *post hoc* test (*p<0.05, **p<0.01, ***p<0.001).
(1.07 MB PDF)

Figure S5 **Representative immunoblot-images used in the quantitative analysis.** The immunoblots for (**A**) soluble SOD1 monomer, (**B**) insoluble SOD1 monomer, (**C**) insoluble high-molecular weight (HMW) SOD1, (**D**) soluble polyubiquitinated proteins, (**E**) insoluble polyubiquitinated proteins, (**F**) soluble p62, (**G**) insoluble p62, (**H**) LC3-I, (**I**) LC3-II, (**J**) insoluble vimentin, (**K**) insoluble GFAP (images for CBB-stained gels), (**L**) soluble GAPDH, and (**M**) insoluble β-tubulin were analyzed. Colored bars drawn above the lanes of each blot indicate the genotypes of samples [wild-type (WT) (black), *Als2* ^−/−^ (orange), *Als2* ^+/+^;*SOD1^H46R^* (blue), *Als2* ^+/−^;*SOD1^H46R^* (green), and *Als2* ^−/−^;*SOD1^H46R^* (red)]. C1 (control sample 1) and C2 (control sample 2) used as internal controls indicate soluble and insoluble fractions from 23 week-old (end-stage) *Als2* ^+/−^;*SOD1^H46R^* mice, respectively. Immunoreactive bands or area indicated as * on the right are quantified using CS Analyzer ver3 (ATTO). In this study, in order to quantify the levels of LC3-II, we used the data obtained form 1% Triton X-100 insoluble fractions (**I**) rather than soluble ones, since the majority of the lipidated form of LC3 (LC3-II) was recovered in this fraction (Figure 4).
(4.07 MB PDF)

Figure S6 **Quantitative analysis of SOD1, ubiquitin, p62, LC3, vimentin, and GFAP in the spinal cord.** (**A**) Quantitation of soluble (1% Triton X-soluble) monomeric SOD1 (SOD1_mono; upper panel), insoluble (1% Triton X-insoluble/5% SDS-soluble) monomeric SOD1 (SOD_mono; middle panel), and insoluble high molecular-weight SOD1 (SOD1_HMW; lower panel). (**B**) Quantitation of soluble (upper panel) and insoluble (lower panel) polyubiquitinated proteins (ubiquitin_HMW). (**C**) Quantitation of soluble (upper panel) and insoluble (lower panel) p62. (**D**) Quantitation of LC3-I (upper panel) and LC3-II (upper panel). (**E**) Quantitation of insoluble vimentin (upper panel) and insoluble GFAP (lower panel). A total of 80 animals [4 animals×4 time-points (8, 12, 16, 20 weeks)×5 genotypes] were used. The soluble and insoluble fractions were prepared from the lumbo-sacral cord of each animal. Densitometric data for immunoreative signals in soluble and insoluble fractions were normalized by the levels of GAPDH and β-tubulin, respectively. Values are mean ± SEM (n = 4) in an arbitrary unit relative to 8 week-old wild-type mice except for the case of SOD1, in which an arbitrary unit relative to *Als2* ^+/+^;*SOD1^H46R^* mice is used. Statistical significance is evaluated by ANOVA with Bonferroni's *post hoc* test. Only the significant differences between *Als2* ^−/−^;*SOD1^H46R^* and the rest of genotypes are shown (*p<0.05, **p<0.01, ***p<0.001).
(0.55 MB PDF)

Figure S7 **SOD1^H46R^ expression causes a progressive accumulation of insoluble SOD1, ubiquitin, and p62 in the brainstem, cerebellum, and spinal cord, but not in the cortex in mice.** Western blot analysis of the levels of (**A**) SOD1, (**B**) p62, and (**C**) ubiquitin in the cortex, brainstem, cerebellum, cervical cord, and lumbo-sacral cord from 8, 12, 16, and 20 week-old mice with four distinct genotypes; wild-type (*Als2* ^+/+^), *Als2* ^+/+^;*SOD1^H46R^*, *Als2* ^+/−^;*SOD1^H46R^*, and *Als2* ^−/−^;*SOD1^H46R^*. Two fractions; 1% Triton X-soluble fraction (TX-soluble; left panels) and 1% Triton X-insoluble/5% SDS-soluble fraction (TX-insoluble; right panels) were analyzed. SOD1_mono and SOD1_HMW represent monomeric and high molecular-weight (aggregated) forms of SOD1, respectively. Ub_mono and Ub_HMW represent monomeric ubiquitin and the polyubiquitinated proteins, respectively. The positions of size-markers are shown on the left (**A**, **C**).
(3.74 MB PDF)

Figure S8 **No obvious SOD1-positive inclusions are observed in the spinal cord of SOD1^H46R^-expressing early-symptomatic mice.** (**A**) Macro-view for the composite images for the transverse section of the lumbar spinal cord (L4–L5) double-immunostained with MAP2 (green) and SOD1 (red) from 16-week-old wild-type (WT; left), 16-week-old *Als2* ^+/+^;*SOD1^H46R^* (middle), and 16-week-old *Als2* ^−/−^;*SOD1^H46R^* (right) mice. The nuclei were counterstained with DAPI (Blue). Scale bar = 100 µm. (**B**) Representative images of double immunostaining with MAP2 (1st column, green) and SOD1 (2nd column, red) for the ventral horn of the lumbar spinal cord (L4–L5) from 16-week-old wild-type (WT; upper row), 16-week-old *Als2* ^+/+^;*SOD1^H46R^* (middle row), and 16-week-old *Als2* ^−/−^;*SOD1^H46R^* (lower row) mice. The nuclei were counterstained with DAPI (3rd column, blue). Scale bar = 100 µm. Scale bar = 20 µm.
(1.03 MB PDF)

Figure S9 **ALS2 is colocalized with autophagosomal and endosomal proteins onto perinuclear vesicular compartments in HeLa cells.** (**A**) Colocalization of ectopically expressed ALS2 (green) with endogenous p62 (red; upper), LAMP2 (red; middle), or EEA1 (red; lower) in HeLa cells. Right columns display the merged images. (**B**) Ectopically expressed EGFP-LC3 (green) and ALS2 (red) are both partially colocalized with either endogenous p62 (blue; upper row), LAMP2 (blue; middle row), or EEA1 (blue; lower row) onto perinuclear vesicular compartments in HeLa cells. Third and fifth columns display the merged images for double (LC3 and ALS2) and triple immunostainings, respectively. It is notable that ectopic expression of EGFP-LC3 enhances the vesicular localization of ALS2 in HeLa cells. Scale bars = 10 µm.
(0.46 MB PDF)

Figure S10 **ALS2 enhances the autophagic clearance of LC3 in fibroblasts.** (**A**) Western blot analysis of the levels of ALS2, p62, and LC3 in primary fibroblasts from *Als2* ^−/−^, wild-type (WT), and *ALS2*-tg (line L6-2) mice. Fibroblasts were incubated in a starvation medium for indicated periods. NT denotes “non-treated”. Equal amount of protein from 1% Triton X-soluble fractions was loaded in each lane, and analyzed by immunoblotting using antibodies as indicated. Alpha (α)-tubulin served as control. Loss of ALS2 lowered the starvation-induced LC3-II degradation, while ALS2 overexpression led to marked enhancement of the LC3-II clearance. (**B**) Colocalization of ectopically expressed EGFP-LC3 (green) with endogenous p62 (red) in fibroblasts from WT (upper), *Als2* ^−/−^ (middle), and *ALS2*-tg (lower) mice. Loss of ALS2 resulted in a higher level of EGFP-LC3-positive puncta/vesicles (green) colocalizing with p62 (middle). It is notable that EGFP-LC3 (green) was recruited to the ALS2-induced enlarged vesicles/vacuoles, which might result from the enhanced endosome fusion by ALS2 overexpression, with a concomitant decrease in the colocalization with p62 (lower). Right columns display the merged images. Scale bars = 10 µm.
(0.40 MB PDF)

Figure S11 **ALS2 regulates the level of LC3 in HeLa cells.** (**A**) Small interfering RNA (siRNA)-mediated suppression of ALS2 results in an increased level of LC3-II in HeLa cells. The cells were treated with 3 independent siRNAs for *ALS2*; ALS2 si-1 (left), ALS2 si-2 (middle), and ALS2 si-3 (right). Scrambled siRNA was used as a control. Total lysates were analyzed by immunoblotting using anti-ALS2 and anti-LC3 antibodies. Alpha-tubulin (α-Tub) served as control. (**B**) Quantitative densitometry for the levels of LC3-II immunoreactive signals shown in **A**. Data were normalized by the levels of α-tubulin (LC3-II/α-tubulin). Values are mean ± SEM (n = 4) in an arbitrary unit relative to control. Statistical significance is evaluated by ANOVA with Bonferroni's *post hoc* test (*p<0.05, ***p<0.001). (**C**) Effect of siRNA-mediated suppression of ALS2 on autophagic flux in HeLa cells. The cells were treated with 4 independent siRNAs for *ALS2* (48 hr), followed by the incubation with or without 12.5 µM chloroquine (CQ) for another 8 hr. Scrambled siRNA was used as a control. Total lysates were analyzed by immunoblotting using anti-ALS2 and anti-LC3 antibodies. Alpha-tubulin (α-Tub) served as control. (**D**) Representative images for double immunostainings with LC3 (green) and p62 (red) in HeLa cells. The cells were treated with either ALS2-si1 or control siRNA, followed by a transient starvation (30 min). Right columns display the merged images. Scale bars = 10 µm.
(1.92 MB PDF)

Figure S12 **Loss of ALS2 results in a decreased level of starvation-induced autophagic clearance of p62 in primary spinal neurons.** (**A**–**D**) Representative p62-immunostaining images for primary spinal motor neurons derived from wild-type (WT) (**A** and **C**) and *Als2* ^−/−^ (**B** and **D**) mice. The cells (DIV14) were either left unstarved (0 hr) (**A** and **B**) or starved for 6 hr (**C** and **D**). Scale bars = 10 Î¼m. (**E** and **F**) Signal intensities for the p62-immunoreactivity in randomly selected spinal neurons under unstarved (0 hr; **E**) and starved (6 hr; **F**) conditions. Fluorescent intensities within the digitally-demarcated area corresponding to the cell body of each cell were measured. Signal intensity relative to unit area (y-axis) was calculated and expressed as arbitrary units (AU). The numbers of cells analyzed (x-axis) are as follows; (**E**, unstarved) WT; n = 149, *Als2* ^−/−^; n = 127, and (**F**, starved) WT; n = 123, *Als2* ^−/−^; n = 139. Blue diamonds and red squares represent data for WT and *Als2* ^−/−^ cells, respectively.
(2.09 MB PDF)

Table S1 **Genotypes of offsprings produced by crossing male** ***Als2*** **^+/−^;** ***SOD1^H46R^*** **and female** ***Als2*** **^+/−^mice.**
(0.04 MB PDF)
