# Supplementary figures and images for: Loss of ALS2/Alsin Exacerbates Motor Dysfunction in a SOD1H46R-Expressing Mouse ALS Model by Disturbing Endolysosomal Trafficking
Source: PLoS One. 2010 Mar 22;5(3):e9805. doi: 10.1371/journal.pone.0009805 (PMC2842444; doi:10.1371/journal.pone.0009805)

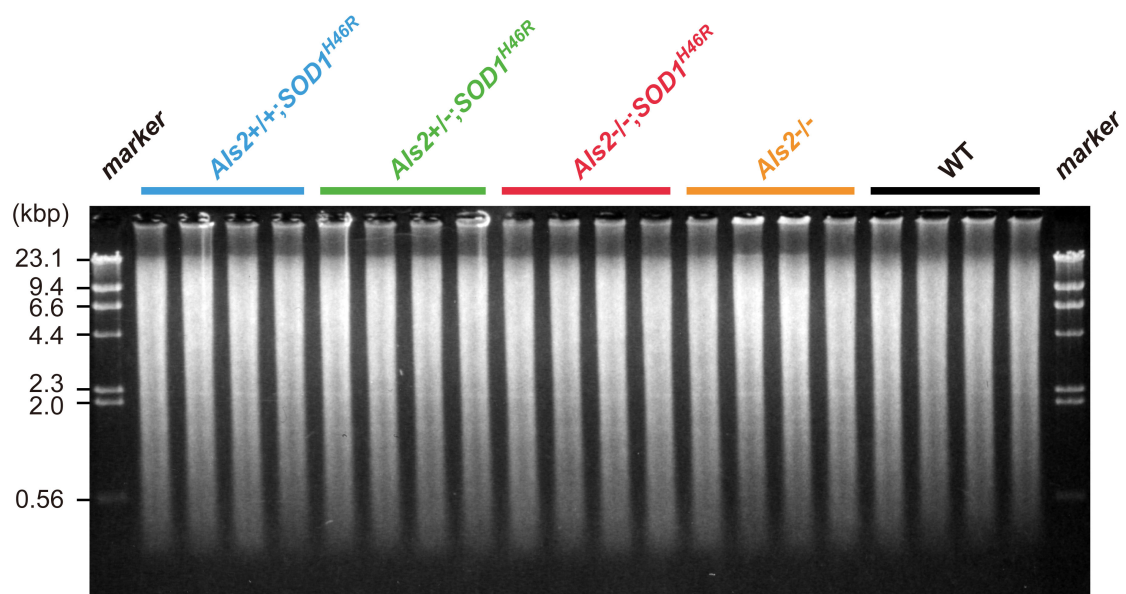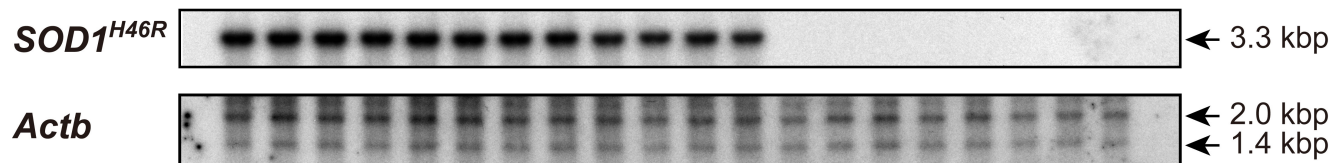

Supplement: Figure S1 — Copy numbers of the transgene in human SOD1H46R transgenic mice on different Als2 genotypes used were comparable. (Upper panel) Image for the ethidium bromide-stained mouse genomic DNA digested with FbaI. Mice with five different genotypes [Als2 +/+;SOD1H46R (blue), Als2 +/−;SOD1H46R (green), Als2 −/−;SOD1H46R (red), Als2 −/− (orange), and wild-type (WT) (black), (each n = 4)] were analyzed. Equal amount of genomic DNA (2 µg) was loaded and separated by agarose gel electrophoresis. The positions of size-markers are shown on the left. (Middle panel) Southern blot analysis of the mouse genomic DNA. The FbaI blot was probed with the radio-labeled human genomic DNA fragment of the SOD1 gene. A 3.3 kbp of the restriction fragment originating from human SOD1H46R transgene was specifically detected. (Lower panel) As a control, the FbaI blot was re-probed with the radio-labeled mouse Actb (β-actin) cDNA, detecting two fragments originating from mouse endogenous Actb gene (2.0 and 1.4 kbp). (1.18 MB PDF) [file pone.0009805.s001.pdf]

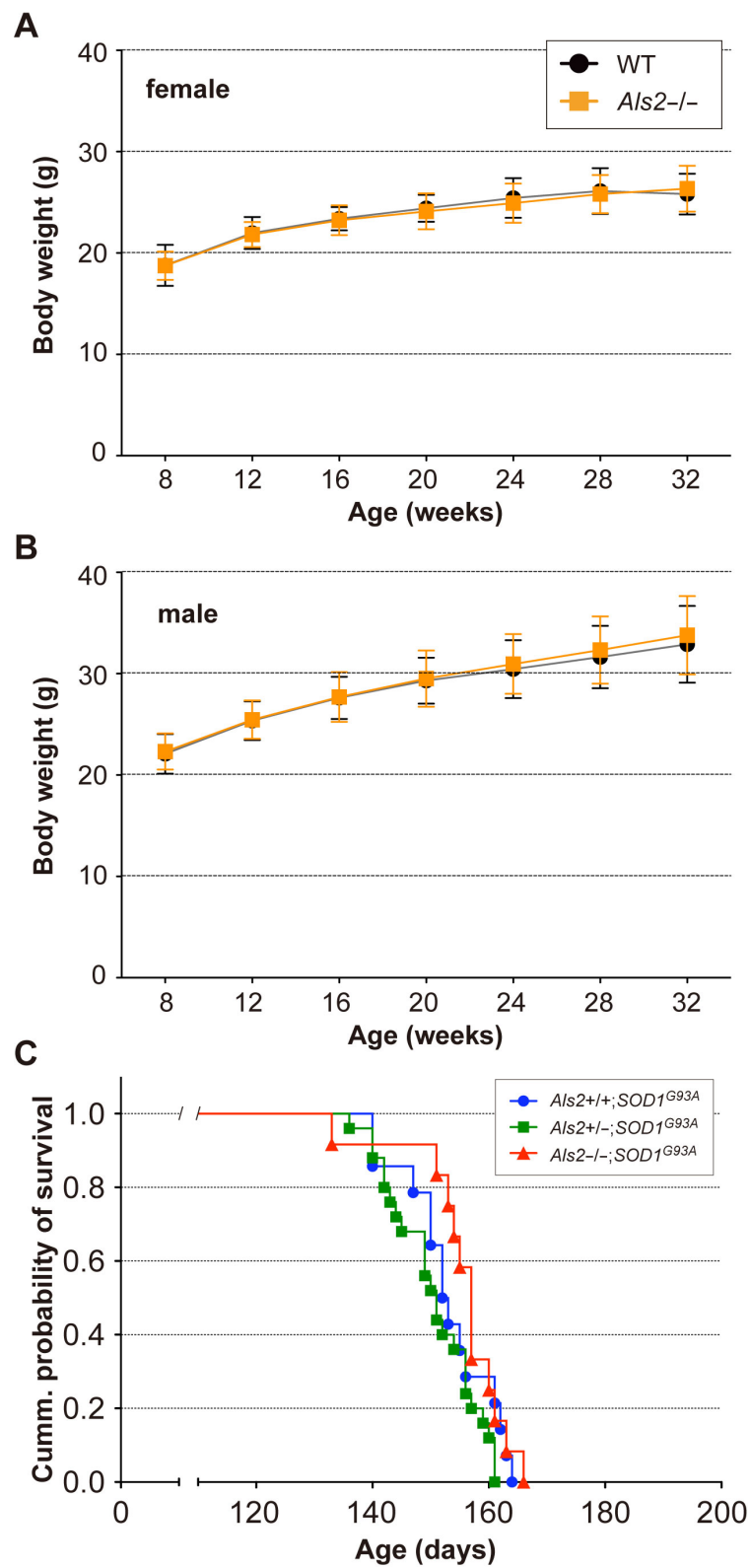

Supplement: Figure S2 — Growth curves for Als2 −/− mice and effect of ALS2 loss on survival in SOD1G93A mice. (A) Growth curves for female mice [wild-type (WT) (black circle; n = 16–36) and Als2 −/− (orange square; n = 32–56)], and (B) for male mice [WT (n = 42–49) and Als2 −/− (n = 46–55)]. (A–B) There were no differences in the mean values between WT and Als2 −/− mice at any ages. Values are mean ± SD. Statistical significance is evaluated by ANOVA with Tukey's post hoc test. (C) Survival curves for Als2 +/+;SOD1G93A [blue circle; n = 14 (female; n = 2, male; n = 12)], Als2 +/−;SOD1G93A [green square; n = 25 (female; n = 13, male; n = 12)], and Als2 −/−;SOD1G93A [red triangle; n = 12 (female; n = 8, male; n = 4)]. Kaplan-Meier analysis identified significant difference between Als2 +/−;SOD1G93A and Als2 −/−;SOD1G93A (Log-rank test; p = 0.0479), while no significance between Als2 +/+;SOD1G93A and Als2 −/−;SOD1G93A was detected. (0.36 MB PDF) [file pone.0009805.s002.pdf]

**A**

## Human full-length *ALS2*-tg construct (~12 kb)

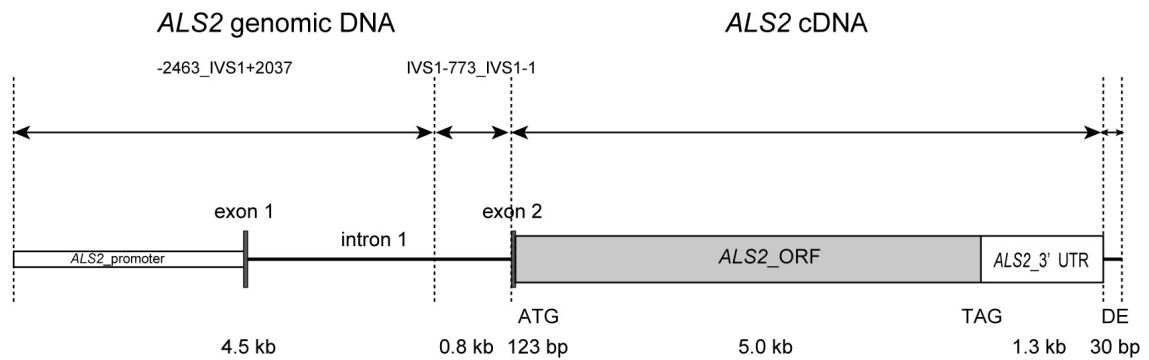**B**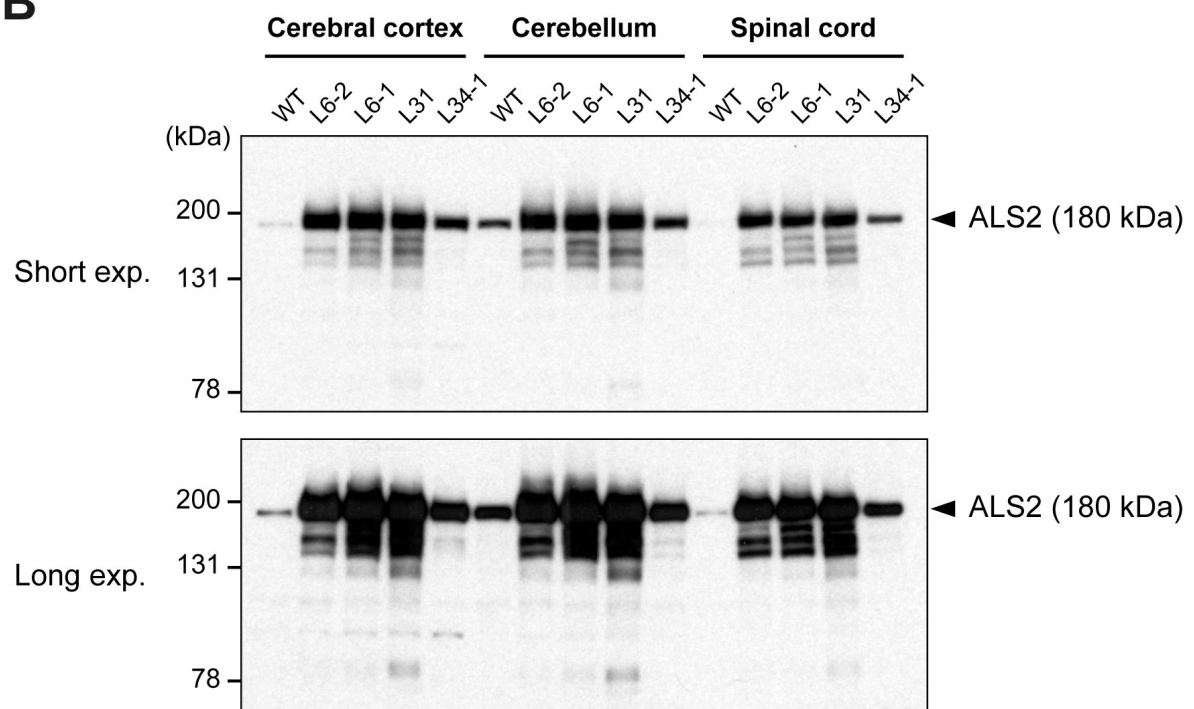

Supplement: Figure S3 — Generation of human full-length ALS2 expressing mice. (A) Schematic representation of the ALS2 transgenic construct. Human full-length ALS2 transcript is expressed under the control of the human ALS2 promoter. (B) Western blot analysis of ALS2 expression in cerebral cortex, cerebellum, and spinal cord from wild-type (WT) and 4 independent ALS2-tg lines; L6-2, L6-1, L31, and L34-1. Equal amount of protein from 1% Triton X-soluble fractions (5 Î¼g) was loaded in each lane, and anti-ALS2 polyclonal antibody (HPF1-680) was used to probe ALS2 (180 kDa) as indicated on the right. Upper and lower panels represent images for short and long exposures, respectively. The positions of size-markers are shown on the left. (0.29 MB PDF) [file pone.0009805.s003.pdf]

**A**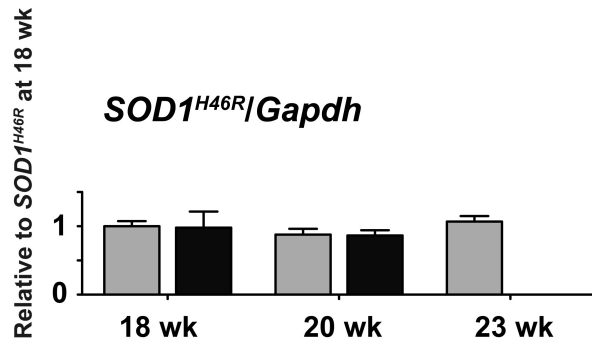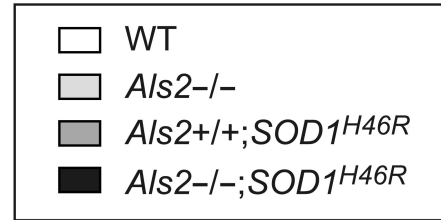**B**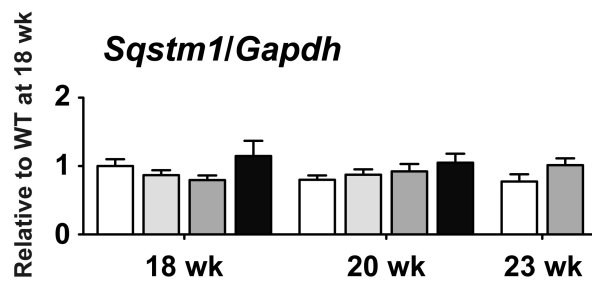**E**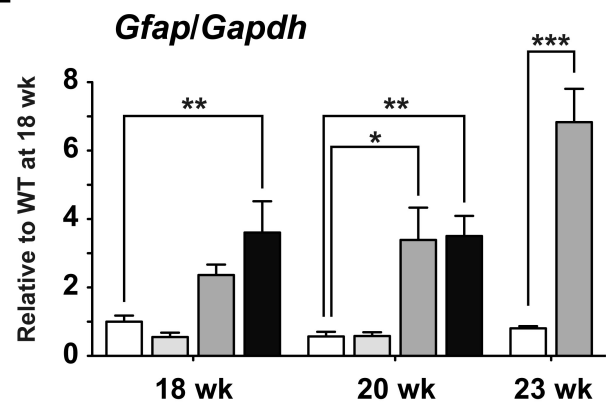**C**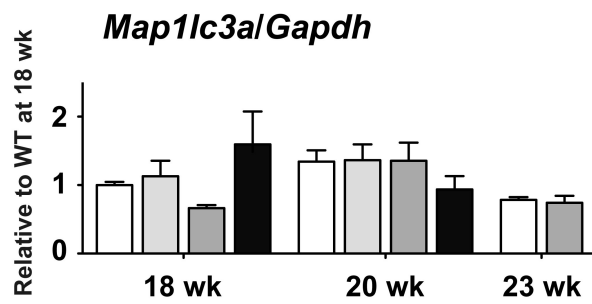**F**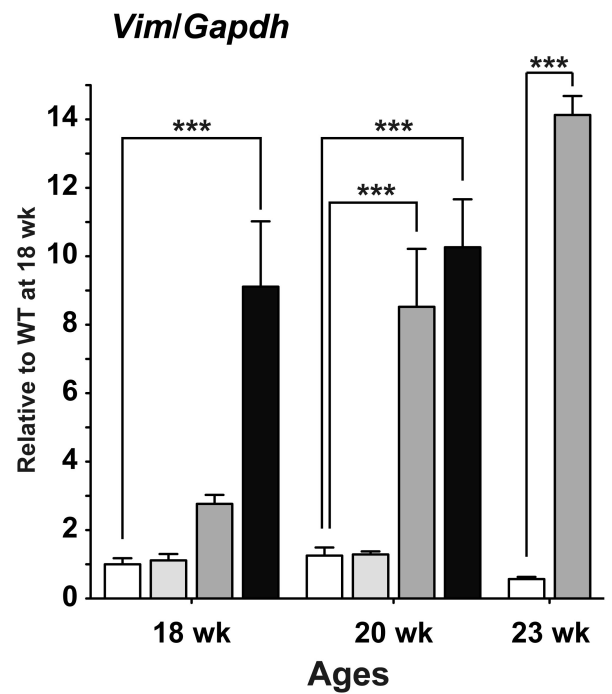**D**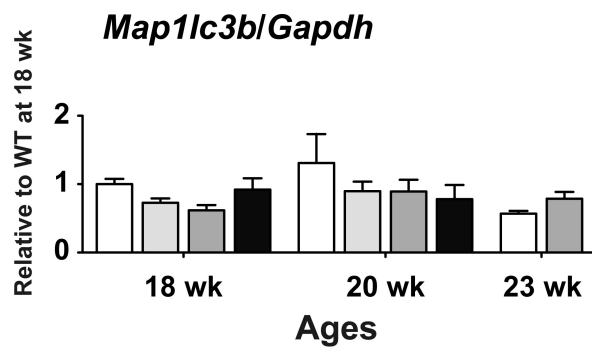

Supplement: Figure S4 — Quantitative analysis of the transcripts in the spinal cord. The expression levels of (A) the human SOD1H46R transgene, (B) Sqstm1 (p62), (C) Map1lc3a (LC3A), (D) Map1lc3b (LC3B), (E) Gfap, and (F) Vim (vimentin) genes, which are normalized by the level of Gapdh, in the lumbo-sacral cord from 18, 20, and 23 week-old mice with four distinct genotypes; wild-type (WT), Als2 −/−, Als2 +/+;SOD1H46R, and Als2 −/−;SOD1H46R are analyzed. Values are mean ± SEM (n = 3–5) in an arbitrary unit relative to 18 week-old wild-type mice except for the SOD1H46R expression in which values relative to 18 week-old Als2 +/+;SOD1H46R mice are shown. Statistical significance is evaluated by ANOVA with Bonferroni's post hoc test (*p<0.05, **p<0.01, ***p<0.001). (1.07 MB PDF) [file pone.0009805.s004.pdf]

**A**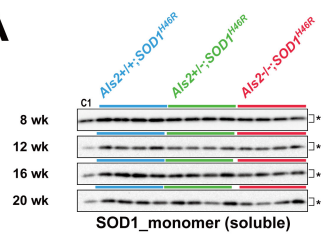**B**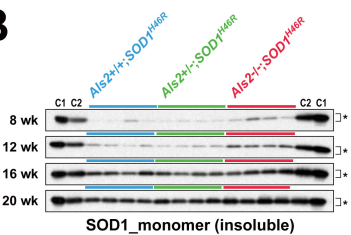**C**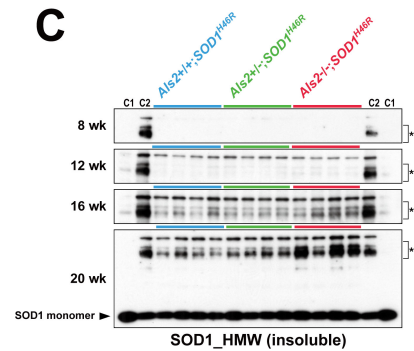**D**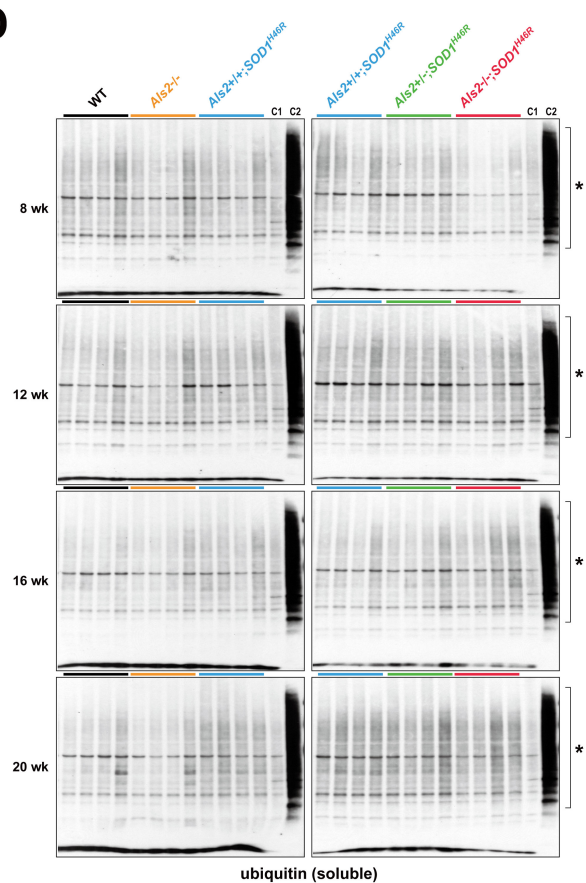**E**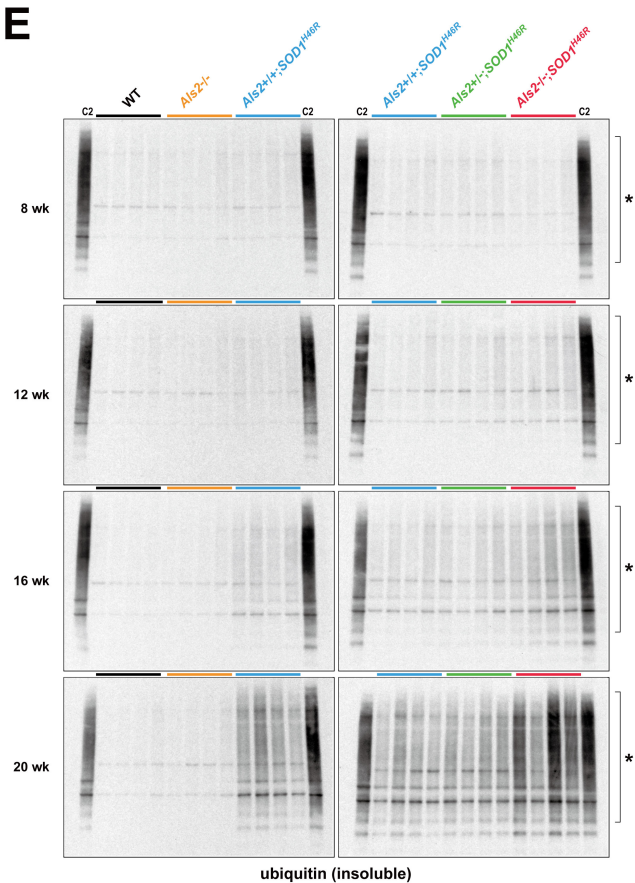

**F**

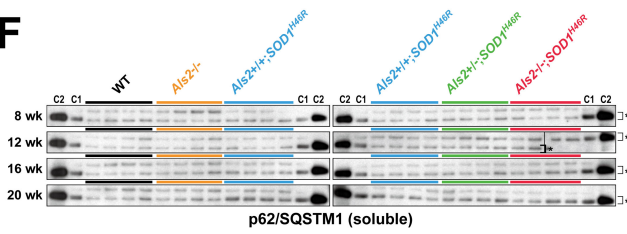

**G**

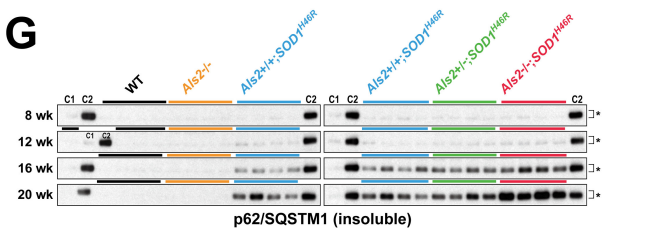

**H**

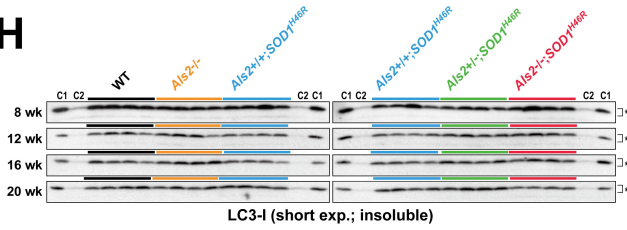

**I**

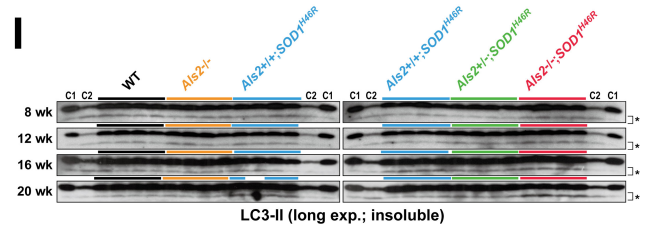

**J**

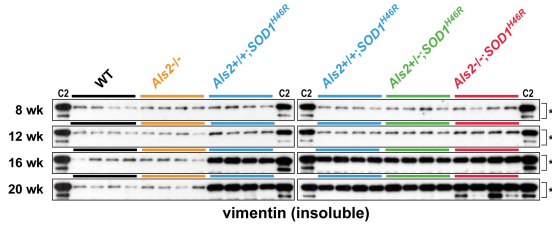

**K**

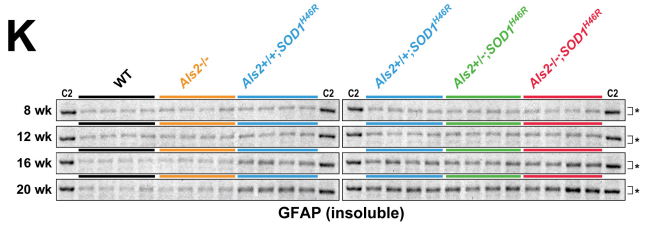

**L**

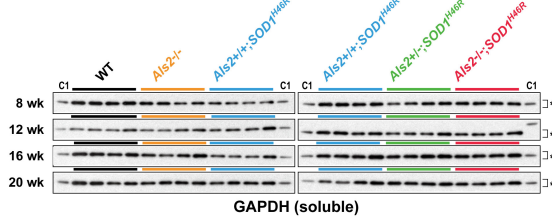

**M**

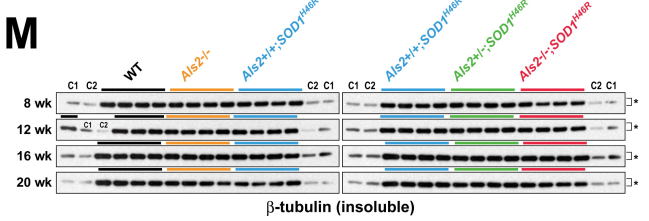

Supplement: Figure S5 — Representative immunoblot-images used in the quantitative analysis. The immunoblots for (A) soluble SOD1 monomer, (B) insoluble SOD1 monomer, (C) insoluble high-molecular weight (HMW) SOD1, (D) soluble polyubiquitinated proteins, (E) insoluble polyubiquitinated proteins, (F) soluble p62, (G) insoluble p62, (H) LC3-I, (I) LC3-II, (J) insoluble vimentin, (K) insoluble GFAP (images for CBB-stained gels), (L) soluble GAPDH, and (M) insoluble β-tubulin were analyzed. Colored bars drawn above the lanes of each blot indicate the genotypes of samples [wild-type (WT) (black), Als2 −/− (orange), Als2 +/+;SOD1H46R (blue), Als2 +/−;SOD1H46R (green), and Als2 −/−;SOD1H46R (red)]. C1 (control sample 1) and C2 (control sample 2) used as internal controls indicate soluble and insoluble fractions from 23 week-old (end-stage) Als2 +/−;SOD1H46R mice, respectively. Immunoreactive bands or area indicated as * on the right are quantified using CS Analyzer ver3 (ATTO). In this study, in order to quantify the levels of LC3-II, we used the data obtained form 1% Triton X-100 insoluble fractions (I) rather than soluble ones, since the majority of the lipidated form of LC3 (LC3-II) was recovered in this fraction (Figure 4). (4.07 MB PDF) [file pone.0009805.s005.pdf]

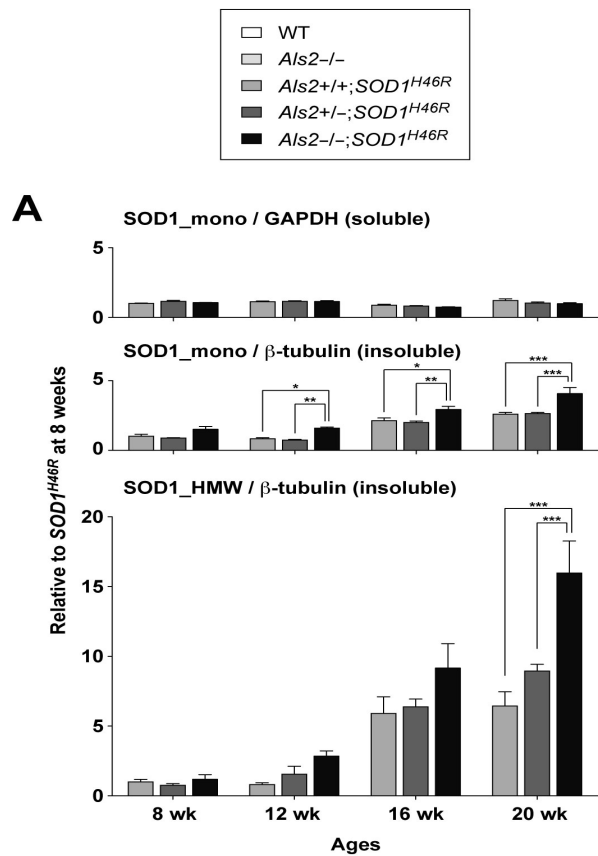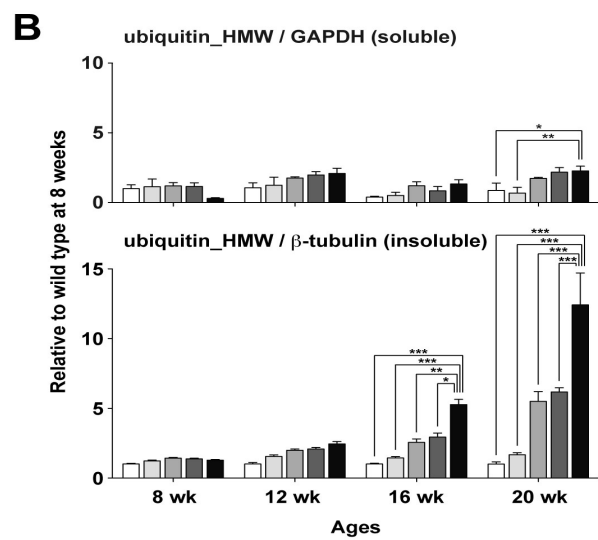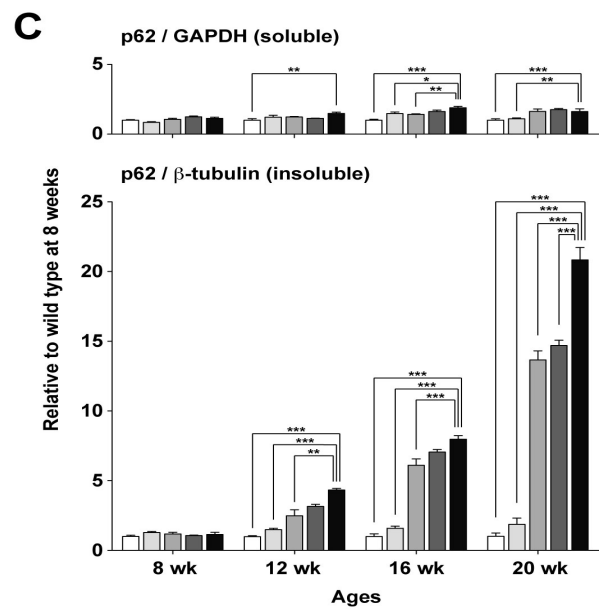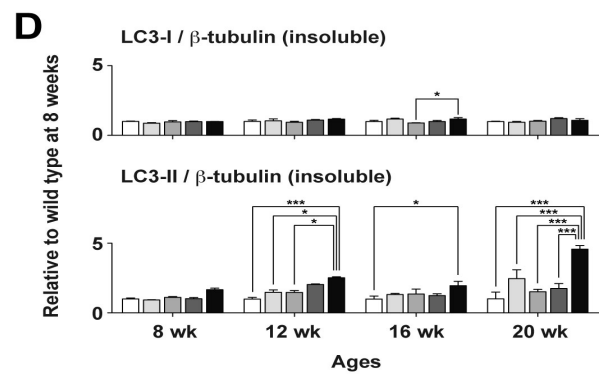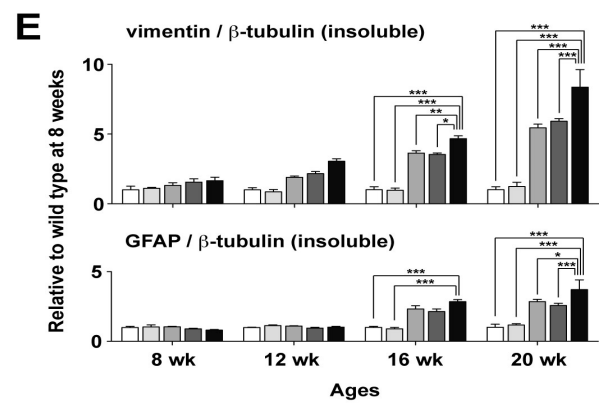

Supplement: Figure S6 — Quantitative analysis of SOD1, ubiquitin, p62, LC3, vimentin, and GFAP in the spinal cord. (A) Quantitation of soluble (1% Triton X-soluble) monomeric SOD1 (SOD1_mono; upper panel), insoluble (1% Triton X-insoluble/5% SDS-soluble) monomeric SOD1 (SOD_mono; middle panel), and insoluble high molecular-weight SOD1 (SOD1_HMW; lower panel). (B) Quantitation of soluble (upper panel) and insoluble (lower panel) polyubiquitinated proteins (ubiquitin_HMW). (C) Quantitation of soluble (upper panel) and insoluble (lower panel) p62. (D) Quantitation of LC3-I (upper panel) and LC3-II (upper panel). (E) Quantitation of insoluble vimentin (upper panel) and insoluble GFAP (lower panel). A total of 80 animals [4 animals×4 time-points (8, 12, 16, 20 weeks)×5 genotypes] were used. The soluble and insoluble fractions were prepared from the lumbo-sacral cord of each animal. Densitometric data for immunoreative signals in soluble and insoluble fractions were normalized by the levels of GAPDH and β-tubulin, respectively. Values are mean ± SEM (n = 4) in an arbitrary unit relative to 8 week-old wild-type mice except for the case of SOD1, in which an arbitrary unit relative to Als2 +/+;SOD1H46R mice is used. Statistical significance is evaluated by ANOVA with Bonferroni's post hoc test. Only the significant differences between Als2 −/−;SOD1H46R and the rest of genotypes are shown (*p<0.05, **p<0.01, ***p<0.001). (0.55 MB PDF) [file pone.0009805.s006.pdf]

## SOD1

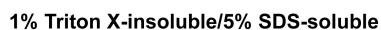

**p62**

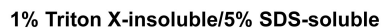

C

# Ubiquitin

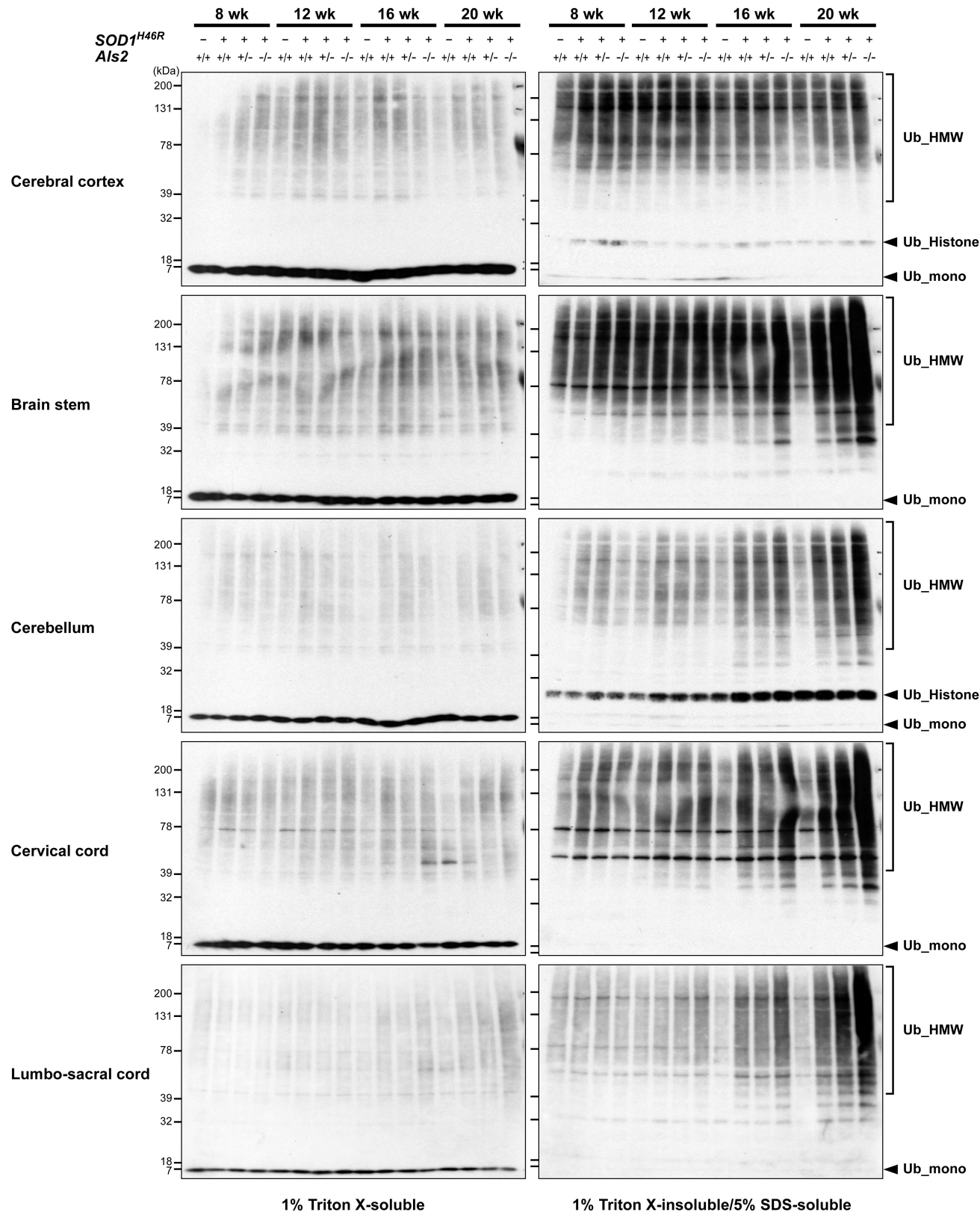

Supplement: Figure S7 — SOD1H46R expression causes a progressive accumulation of insoluble SOD1, ubiquitin, and p62 in the brainstem, cerebellum, and spinal cord, but not in the cortex in mice. Western blot analysis of the levels of (A) SOD1, (B) p62, and (C) ubiquitin in the cortex, brainstem, cerebellum, cervical cord, and lumbo-sacral cord from 8, 12, 16, and 20 week-old mice with four distinct genotypes; wild-type (Als2 +/+), Als2 +/+;SOD1H46R, Als2 +/−;SOD1H46R, and Als2 −/−;SOD1H46R. Two fractions; 1% Triton X-soluble fraction (TX-soluble; left panels) and 1% Triton X-insoluble/5% SDS-soluble fraction (TX-insoluble; right panels) were analyzed. SOD1_mono and SOD1_HMW represent monomeric and high molecular-weight (aggregated) forms of SOD1, respectively. Ub_mono and Ub_HMW represent monomeric ubiquitin and the polyubiquitinated proteins, respectively. The positions of size-markers are shown on the left (A, C). (3.74 MB PDF) [file pone.0009805.s007.pdf]

**A**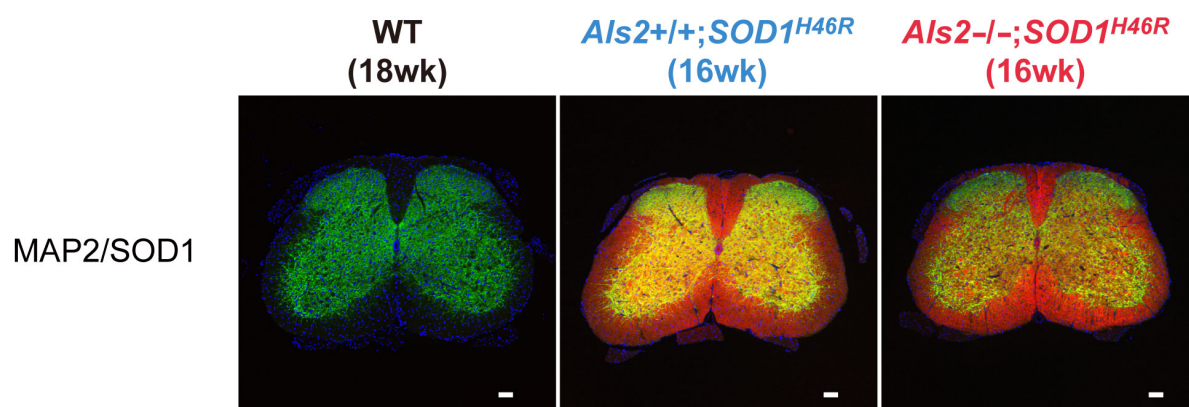**B**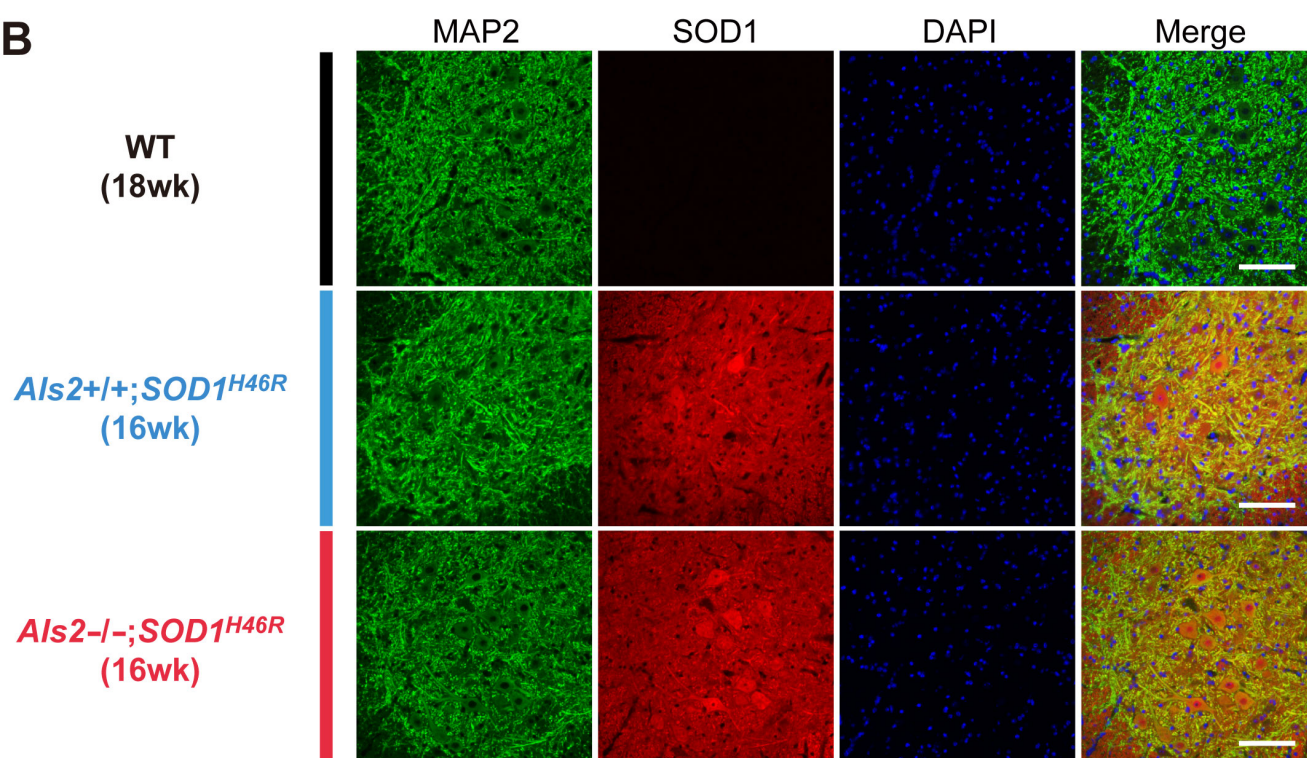

Supplement: Figure S8 — No obvious SOD1-positive inclusions are observed in the spinal cord of SOD1H46R-expressing early-symptomatic mice. (A) Macro-view for the composite images for the transverse section of the lumbar spinal cord (L4–L5) double-immunostained with MAP2 (green) and SOD1 (red) from 16-week-old wild-type (WT; left), 16-week-old Als2 +/+;SOD1H46R (middle), and 16-week-old Als2 −/−;SOD1H46R (right) mice. The nuclei were counterstained with DAPI (Blue). Scale bar = 100 µm. (B) Representative images of double immunostaining with MAP2 (1st column, green) and SOD1 (2nd column, red) for the ventral horn of the lumbar spinal cord (L4–L5) from 16-week-old wild-type (WT; upper row), 16-week-old Als2 +/+;SOD1H46R (middle row), and 16-week-old Als2 −/−;SOD1H46R (lower row) mice. The nuclei were counterstained with DAPI (3rd column, blue). Scale bar = 100 µm. Scale bar = 20 µm. (1.03 MB PDF) [file pone.0009805.s008.pdf]

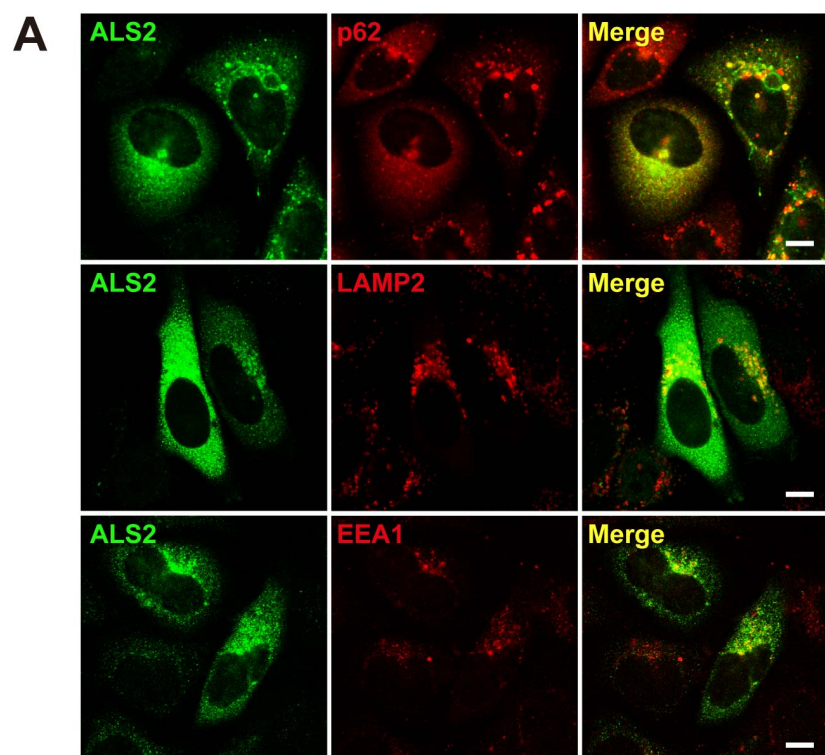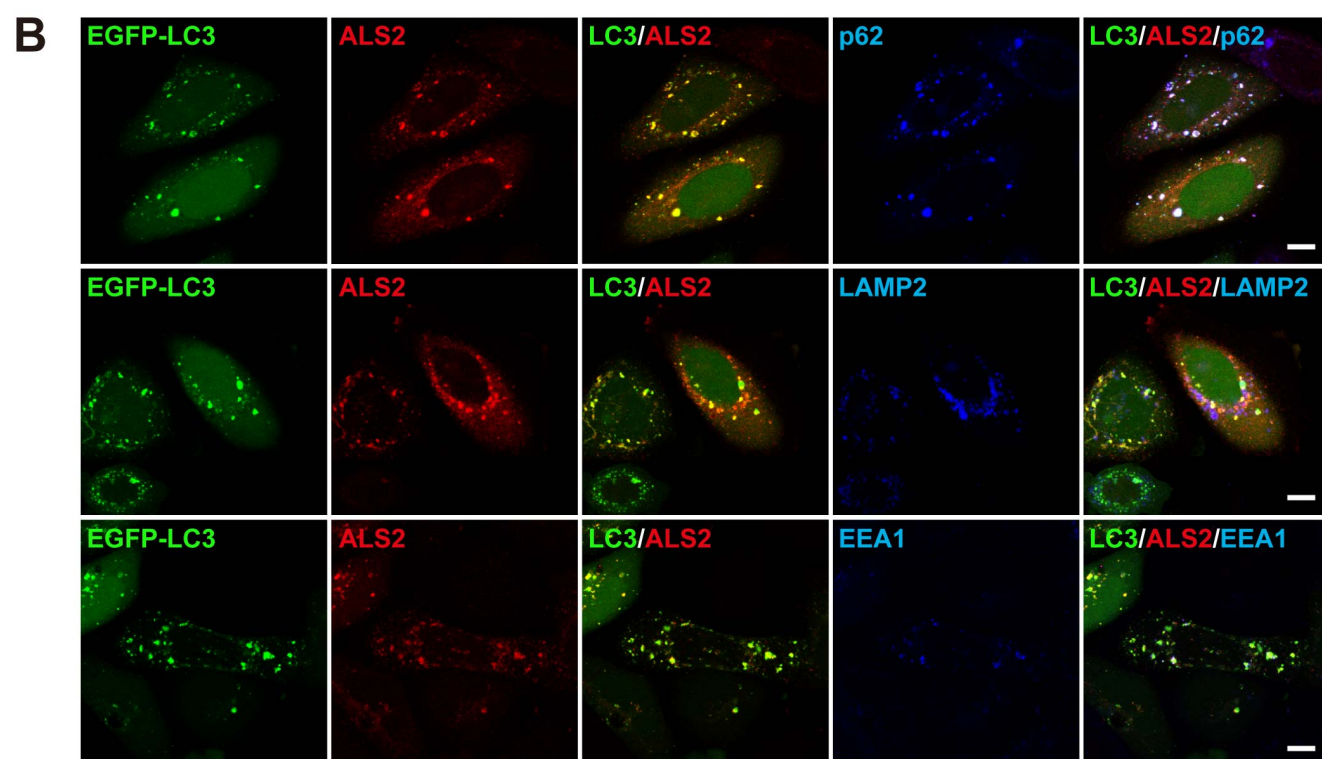

Supplement: Figure S9 — ALS2 is colocalized with autophagosomal and endosomal proteins onto perinuclear vesicular compartments in HeLa cells. (A) Colocalization of ectopically expressed ALS2 (green) with endogenous p62 (red; upper), LAMP2 (red; middle), or EEA1 (red; lower) in HeLa cells. Right columns display the merged images. (B) Ectopically expressed EGFP-LC3 (green) and ALS2 (red) are both partially colocalized with either endogenous p62 (blue; upper row), LAMP2 (blue; middle row), or EEA1 (blue; lower row) onto perinuclear vesicular compartments in HeLa cells. Third and fifth columns display the merged images for double (LC3 and ALS2) and triple immunostainings, respectively. It is notable that ectopic expression of EGFP-LC3 enhances the vesicular localization of ALS2 in HeLa cells. Scale bars = 10 µm. (0.46 MB PDF) [file pone.0009805.s009.pdf]

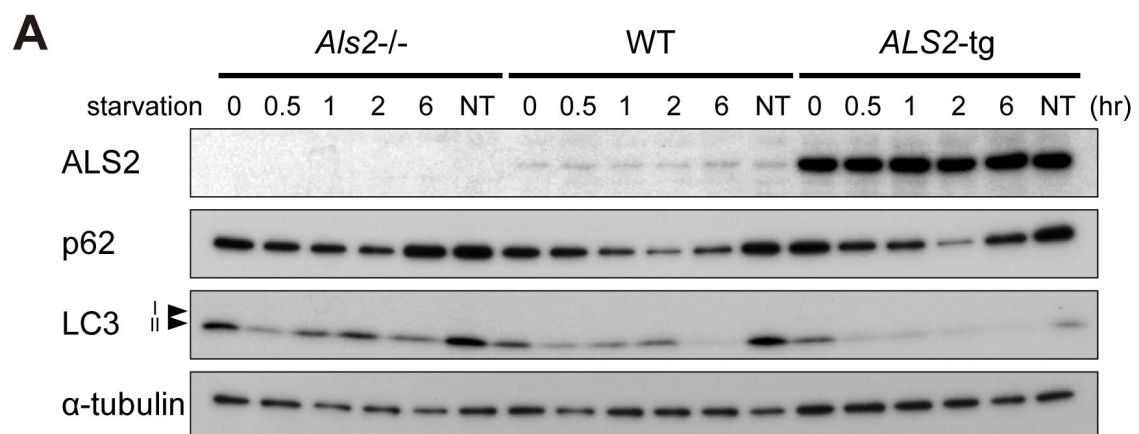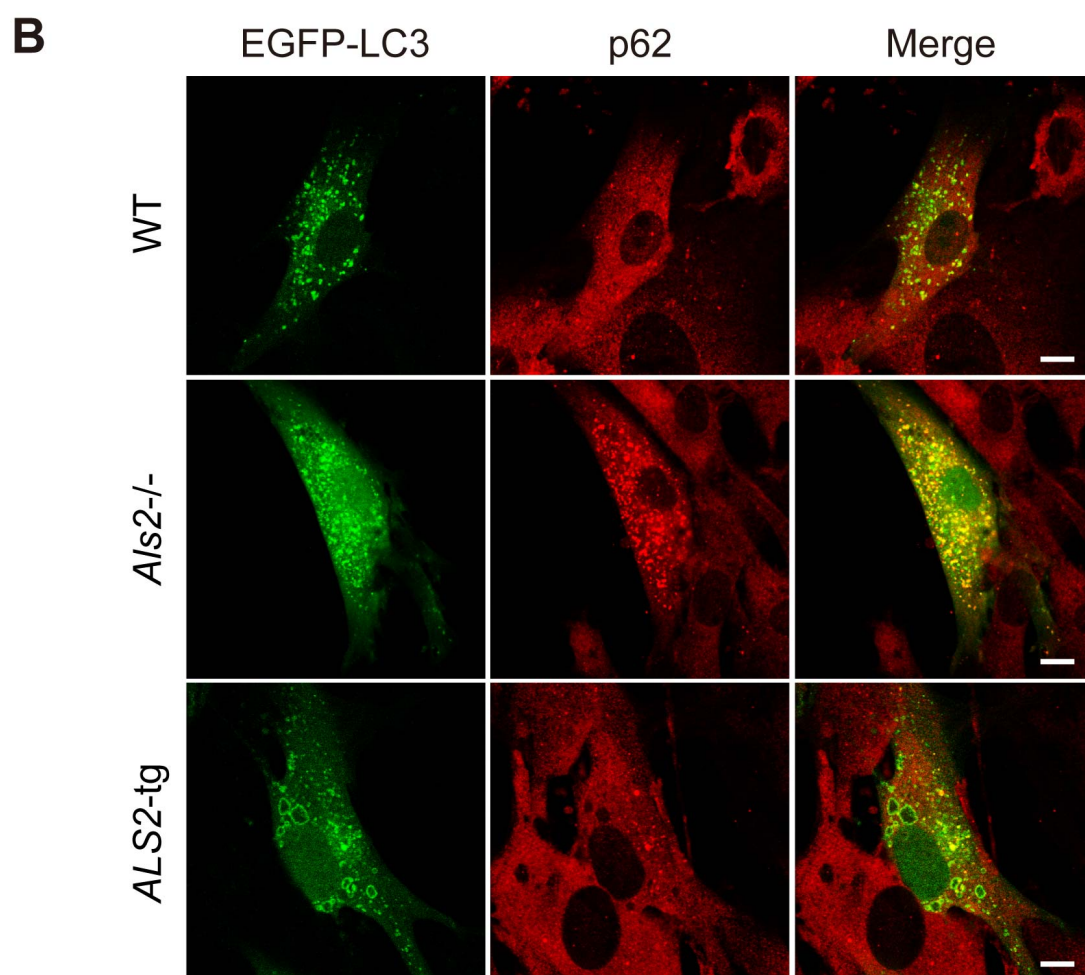

Supplement: Figure S10 — ALS2 enhances the autophagic clearance of LC3 in fibroblasts. (A) Western blot analysis of the levels of ALS2, p62, and LC3 in primary fibroblasts from Als2 −/−, wild-type (WT), and ALS2-tg (line L6-2) mice. Fibroblasts were incubated in a starvation medium for indicated periods. NT denotes “non-treated”. Equal amount of protein from 1% Triton X-soluble fractions was loaded in each lane, and analyzed by immunoblotting using antibodies as indicated. Alpha (α)-tubulin served as control. Loss of ALS2 lowered the starvation-induced LC3-II degradation, while ALS2 overexpression led to marked enhancement of the LC3-II clearance. (B) Colocalization of ectopically expressed EGFP-LC3 (green) with endogenous p62 (red) in fibroblasts from WT (upper), Als2 −/− (middle), and ALS2-tg (lower) mice. Loss of ALS2 resulted in a higher level of EGFP-LC3-positive puncta/vesicles (green) colocalizing with p62 (middle). It is notable that EGFP-LC3 (green) was recruited to the ALS2-induced enlarged vesicles/vacuoles, which might result from the enhanced endosome fusion by ALS2 overexpression, with a concomitant decrease in the colocalization with p62 (lower). Right columns display the merged images. Scale bars = 10 µm. (0.40 MB PDF) [file pone.0009805.s010.pdf]

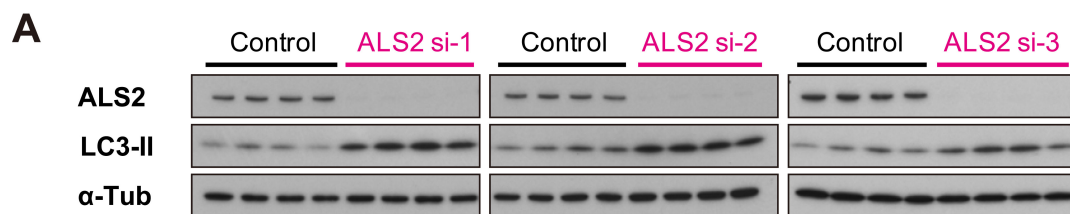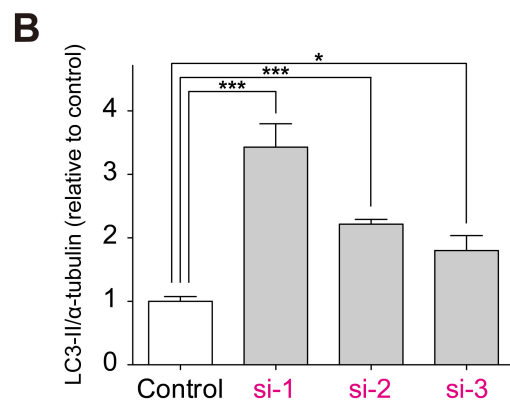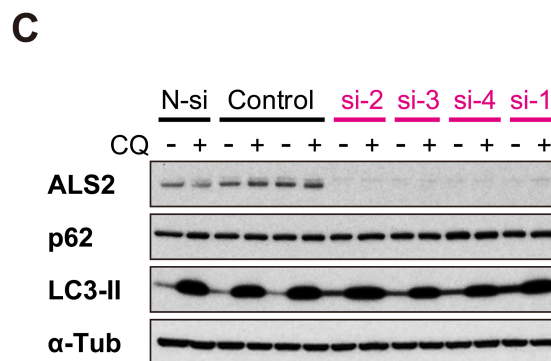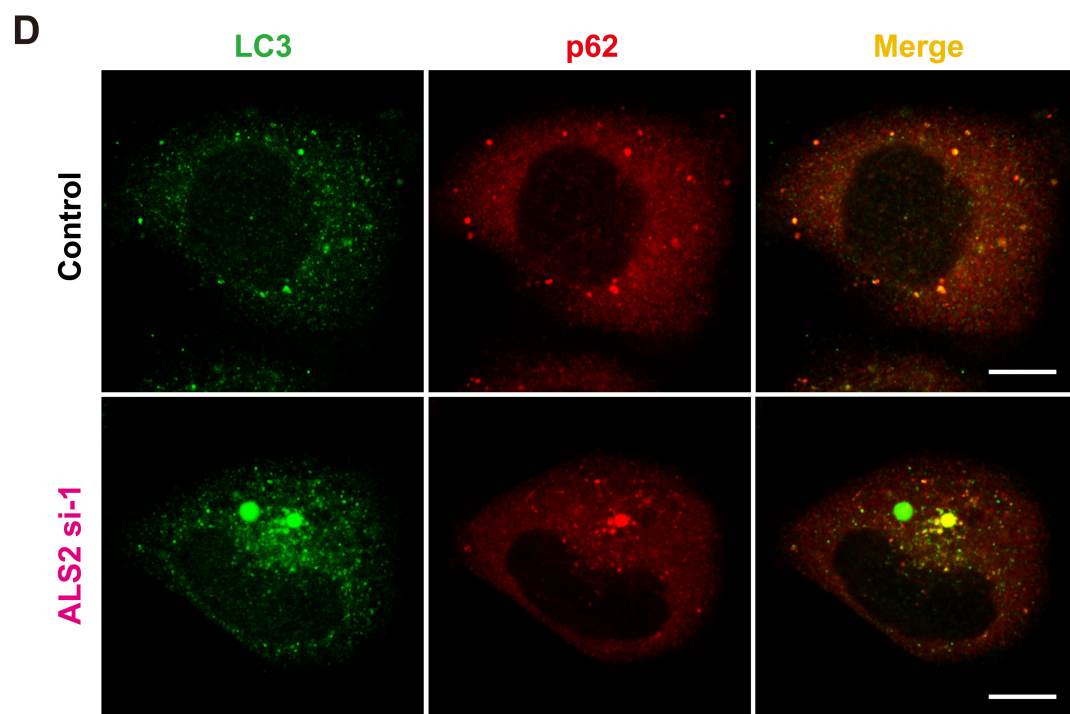

Supplement: Figure S11 — ALS2 regulates the level of LC3 in HeLa cells. (A) Small interfering RNA (siRNA)-mediated suppression of ALS2 results in an increased level of LC3-II in HeLa cells. The cells were treated with 3 independent siRNAs for ALS2; ALS2 si-1 (left), ALS2 si-2 (middle), and ALS2 si-3 (right). Scrambled siRNA was used as a control. Total lysates were analyzed by immunoblotting using anti-ALS2 and anti-LC3 antibodies. Alpha-tubulin (α-Tub) served as control. (B) Quantitative densitometry for the levels of LC3-II immunoreactive signals shown in A. Data were normalized by the levels of α-tubulin (LC3-II/α-tubulin). Values are mean ± SEM (n = 4) in an arbitrary unit relative to control. Statistical significance is evaluated by ANOVA with Bonferroni's post hoc test (*p<0.05, ***p<0.001). (C) Effect of siRNA-mediated suppression of ALS2 on autophagic flux in HeLa cells. The cells were treated with 4 independent siRNAs for ALS2 (48 hr), followed by the incubation with or without 12.5 µM chloroquine (CQ) for another 8 hr. Scrambled siRNA was used as a control. Total lysates were analyzed by immunoblotting using anti-ALS2 and anti-LC3 antibodies. Alpha-tubulin (α-Tub) served as control. (D) Representative images for double immunostainings with LC3 (green) and p62 (red) in HeLa cells. The cells were treated with either ALS2-si1 or control siRNA, followed by a transient starvation (30 min). Right columns display the merged images. Scale bars = 10 µm. (1.92 MB PDF) [file pone.0009805.s011.pdf]

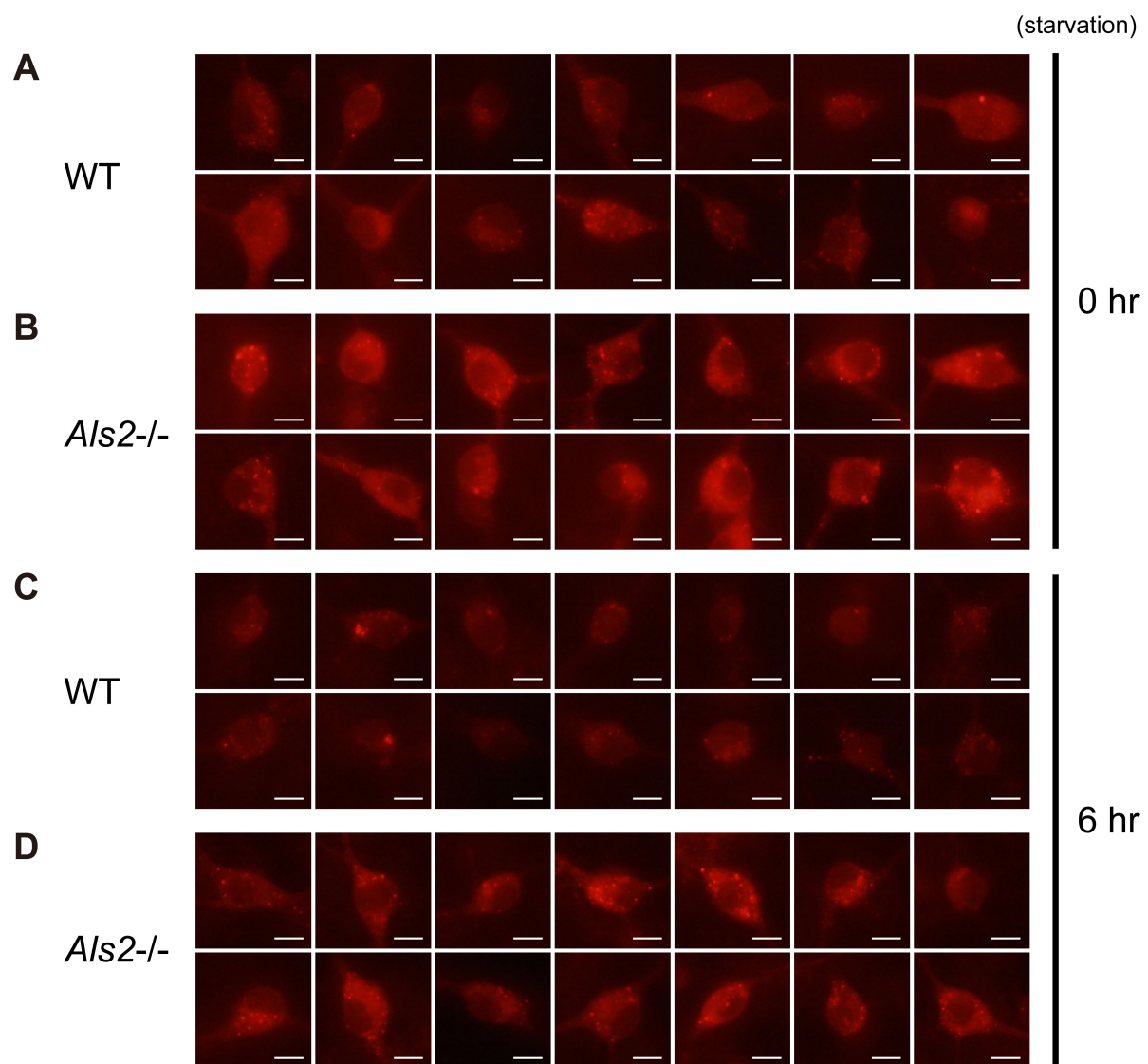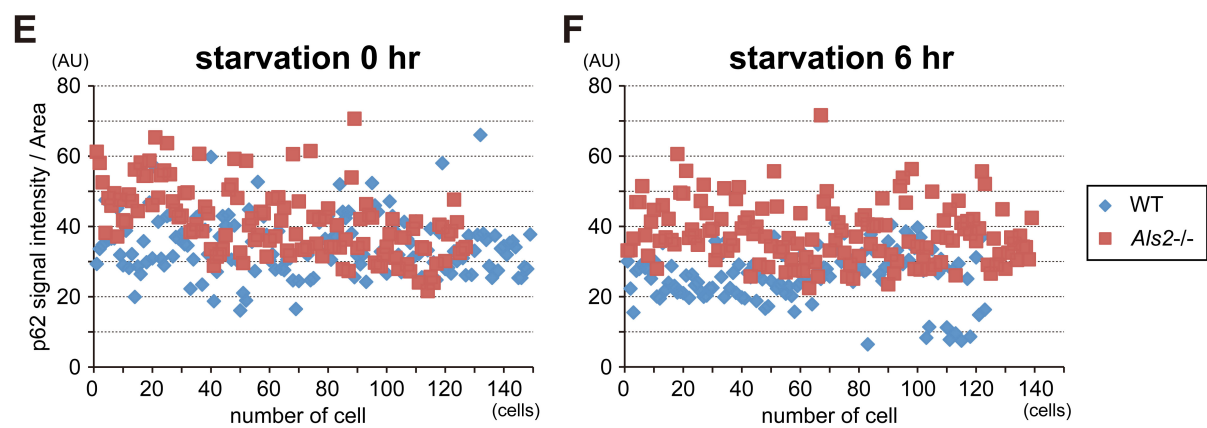

Supplement: Figure S12 — Loss of ALS2 results in a decreased level of starvation-induced autophagic clearance of p62 in primary spinal neurons. (A–D) Representative p62-immunostaining images for primary spinal motor neurons derived from wild-type (WT) (A and C) and Als2 −/− (B and D) mice. The cells (DIV14) were either left unstarved (0 hr) (A and B) or starved for 6 hr (C and D). Scale bars = 10 Î¼m. (E and F) Signal intensities for the p62-immunoreactivity in randomly selected spinal neurons under unstarved (0 hr; E) and starved (6 hr; F) conditions. Fluorescent intensities within the digitally-demarcated area corresponding to the cell body of each cell were measured. Signal intensity relative to unit area (y-axis) was calculated and expressed as arbitrary units (AU). The numbers of cells analyzed (x-axis) are as follows; (E, unstarved) WT; n = 149, Als2 −/−; n = 127, and (F, starved) WT; n = 123, Als2 −/−; n = 139. Blue diamonds and red squares represent data for WT and Als2 −/− cells, respectively. (2.09 MB PDF) [file pone.0009805.s012.pdf]
