# Supplementary material for: Loss of ALS2/Alsin Exacerbates Motor Dysfunction in a SOD1H46R-Expressing Mouse ALS Model by Disturbing Endolysosomal Trafficking
Source: PLoS One. 2010 Mar 22;5(3):e9805. doi: 10.1371/journal.pone.0009805 (PMC2842444; doi:10.1371/journal.pone.0009805)
Supplement: Table S1 — Genotypes of offsprings produced by crossing male Als2 +/−; SOD1H46R and female Als2 +/−mice. (0.04 MB PDF) [file pone.0009805.s013.pdf]

| | ratio | | female observed (n) | male observed (n) | combined (n) |
|---|---|---|---|---|---|
| | expected | observed | | | |
| *Als2*$^{+/+}$ (WT) | 0.125 | 0.119 | 33 | 48 | 81 |
| *Als2*$^{+/-}$ | 0.250 | 0.260 | 80 | 96 | 176 |
| *Als2*$^{-/-}$ | 0.125 | 0.153 | 51 | 53 | 104 |
| *Als2*$^{+/+}$;*SOD1*$^{H46R}$ | 0.125 | 0.112 | 30 | 46 | 76 |
| *Als2*$^{+/-}$;*SOD1*$^{H46R}$ | 0.250 | 0.237 | 85 | 76 | 161 |
| *Als2*$^{-/-}$;*SOD1*$^{H46R}$ | 0.125 | 0.118 | 44 | 36 | 80 |
| total | 1.000 | 1.000 | 323 | 355 | 678 |
